# DNA Physical Properties and Nucleosome Positions Are Major Determinants of HIV-1 Integrase Selectivity

**DOI:** 10.1371/journal.pone.0129427

**Published:** 2015-06-15

**Authors:** Monica Naughtin, Zofia Haftek-Terreau, Johan Xavier, Sam Meyer, Maud Silvain, Yan Jaszczyszyn, Nicolas Levy, Vincent Miele, Mohamed Salah Benleulmi, Marc Ruff, Vincent Parissi, Cédric Vaillant, Marc Lavigne

**Affiliations:** 1 Laboratoire Joliot-Curie, CNRS USR 3010, Ecole Normale Supérieure, Lyon, France; 2 Université de Lyon, Microbiologie Adaptation et Pathogénie, INSA Lyon, CNRS UMR 5240, Lyon, France; 3 Institute for Integrative Biology of the Cell (I2BC), CEA, CNRS UMR9198, Université Paris-Sud, Avenue de la Terrasse, 91198 Gif-sur-Yvette Cedex, France; 4 Institut de Génétique et de Biologie Moléculaire et Cellulaire, Département de Biologie Structurale Intégrative, UDS, CNRS UMR 7104—INSERM U 964, Illkirch, France; 5 Laboratoire de Biométrie et Biologie Evolutive, CNRS UMR 5558, Université Claude Bernard Lyon1, Lyon, France; 6 Laboratoire Microbiologie Fondamentale et Pathogénicité, CNRS UMR 5234, Université Victor Segalen Bordeaux 2, Bordeaux, France; 7 Laboratoire du Physique, CNRS UMR 5672, Ecole Normale Supérieure, Lyon, France; 8 Institut Pasteur, Dpt de Virologie, UMR 3569 CNRS, Paris, France; University of Pittsburgh, UNITED STATES

## Abstract

Retroviral integrases (INs) catalyse the integration of the reverse transcribed viral DNA into the host cell genome. This process is selective, and chromatin has been proposed to be a major factor regulating this step in the viral life cycle. However, the precise underlying mechanisms are still under investigation. We have developed a new *in vitro* integration assay using physiologically-relevant, reconstituted genomic acceptor chromatin and high-throughput determination of nucleosome positions and integration sites, in parallel. A quantitative analysis of the resulting data reveals a chromatin-dependent redistribution of the integration sites and establishes a link between integration sites and nucleosome positions. The co-activator LEDGF/p75 enhanced integration but did not modify the integration sites under these conditions. We also conducted an *in cellulo* genome-wide comparative study of nucleosome positions and human immunodeficiency virus type-1 (HIV-1) integration sites identified experimentally *in vivo*. These studies confirm a preferential integration in nucleosome-covered regions. Using a DNA mechanical energy model, we show that the physical properties of DNA probed by IN binding are important in determining IN selectivity. These novel *in vitro* and *in vivo* approaches confirm that IN has a preference for integration into a nucleosome, and suggest the existence of two levels of IN selectivity. The first depends on the physical properties of the target DNA and notably, the energy required to fit DNA into the IN catalytic pocket. The second depends on the DNA deformation associated with DNA wrapping around a nucleosome. Taken together, these results indicate that HIV-1 IN is a shape-readout DNA binding protein.

## Introduction

Integration of the retroviral genome into the host genome is an essential step of the viral life cycle [1]. Retroviral-encoded integrase is responsible for both 3’end processing and strand transfer of the U3 and U5 ends of the reverse transcribed cDNA, this latter activity being the target of new antiviral strategies [2]. HIV-1 and PFV integrases can also cleave the DNA palindrome formed at the LTR-LTR junction in two- LTR circles. In the case of HIV-1, these cleaved 2LTR circles can act as precursors for integration upon arrest of anti-integrase treatments [3–5].

Retroviral integration is not random, and retroviruses display distinct integration site preferences [6, 7]. At a genomic scale, HIV-1 and other lentiviruses preferentially integrate in the transcribed sequences of active genes [8–12] whereas Moloney murine leukemia virus (MLV) and gammaretroviruses preferentially integrate in transcription start sites [13], enhancers [14], near DNase-1 hypersensitive sites and CpG islands [15]. In addition to the transcription process, other cellular parameters influence IN selectivity including target DNA sequence, chromatin structure, specific host cofactors and the nuclear entry pathway [16].

The role of the target DNA sequence in IN selectivity is mainly local and a weak consensus sequence has been found between integration sites [17, 18]. This sequence is best characterized by its DNA structural properties [17, 18], which are compatible with the strong distortion of the acceptor DNA observed in the crystal structures of the prototype foamy virus (PFV) strand transfer complexes [19] and in the electron-microscopy (EM) structural model of the HIV intasome formed in the presence of its cofactor, the lens-epithelium derived growth factor (LEDGF/p75) [20].

At the chromatin level, HIV-1 integration sites identified in infected cells are positively correlated with both nucleosome positions and specific histone modifications enriched in active genes [15, 21, 22]. However, these correlations were obtained with predicted nucleosome positions and histone marks identified in non-infected cells. The effect of nucleosome positions on HIV-1 IN properties has already been investigated *in vitro*, but there is no study on the effect of histone modifications. *In vitro*, insertion of one viral end (called half-site integration or HSI) is favoured in the nucleosome, with an enrichment of integration sites in widened DNA major grooves facing out of the nucleosome structure [23, 24]. DNA distortions, similar to the one induced by the nucleosomes, also favour the integration process [24–26]. *In vitro*, polynucleosomes (PN) are also preferential IN targets and various parameters affecting their structures influence integration efficiency [27–29]. Interestingly, HSI and full site integration (insertion of two viral ends or FSI) are differently sensitive to chromatin structure [28].

Cellular IN partners constitute another parameter of its selectivity. Among these partners, the transcription co-activator LEDGF/p75 is as a major cofactor of lentiviral INs [30–32]. LEDGF/p75 is required for efficient integration *in vivo*, with very little integration occurring in LEDGF/p75 knockout cells [33–36]. LEDGF/p75 is involved in the selectivity of lentiviral integration and this role is attributed to its DNA and chromatin tethering properties [9, 33–35, 37]. LEDGF/p75 forms a stable complex with HIV-1 IN [38] and structures of this complex alone or interacting with its DNA substrate have been described by electron microscopy (EM) [20]. *In vitro*, LEDGF/p75 enhances both HIV-1 IN 3’processing and strand-transfer activities and regulates its tetramerization [20, 37–43]. *In vitro*, LEDGF/p75 also activates integration into chromatin templates and its PWWP domain is required for this activation, consistent with data obtained *in vivo* [27, 44]. The PWWP domain interacts with both DNA and H3K36 trimethylated histones (H3K36me3) [45–47] and these interactions are suggested to be responsible for the IN selectivity towards transcribed genes, enriched in this histone mark. However, *in vitro*, the direct role of this LEDGF/p75 PWWP-H3K36me3 interaction in IN selectivity hasn’t yet been demonstrated. Interestingly, another family of chromatin binding proteins, the bromo and extra-terminal domain (BET) proteins, interact with Moloney murine leukemia virus (MoMLV) IN and acetylated histones, and are involved in the gammaretrovirus integration selectivity near transcriptional start sites [48–50].

The present study is focused on one parameter of HIV-1 IN selectivity: the nucleosome positions. We chose two different and complementary approaches. The first approach utilizes *in vitro* integration assays in chromatin-reconstituted templates. The major limit of previous integration assays was the use of chromatin templates assembled on artificial repeats of nucleosome positioning sequences [27–29, 51]. The structural properties of these DNA sequences and/or the high stability of nucleosomes assembled on them may affect the IN selectivity. We therefore assembled chromatin on human genomic DNA sequences that should provide more physiological substrates for *in vitro* studies of retroviral integration. We performed an extensive study of nucleosome positions, DNA and IN properties on these chromatin templates that confirmed the existence of two levels of IN selectivity. The second experimental approach corresponds to genomic studies investigating nucleosome occupancy around integration sites identified *in vivo*. This study took advantage of previously published nucleosome positions determined by MNase seq in human cell lines [52, 53] and integration sites identified in infected cells [21, 22]. We also used nucleosome positions predicted with a model that has already been successfully applied to *in vitro* nucleosome positioning [54]. Results obtained by this genomic approach confirm the two levels of IN selectivity identified *in vitro* and the physiological relevance of our new *in vitro* integration assay. We can conclude that two levels of IN site selectivity exist: (1) the integration-site specific energy required for deforming the target DNA within the enzymatic complex; (2) favourable DNA deformation resulting from nucleosome wrapping.

## Materials and Methods

### Cloning of HIV integration sites and chromatin reconstitution

Genomic sequences CL529183, CL529481 and CL528939 [11] and DX598014 [10] of 1.2 kb and containing HIV integration sites identified *in vivo* were amplified from a genomic DNA library (Invitrogen), and cloned into the *Xho* I / *Cla* I sites of the plasmid pBSK-zeo. DNA fragments were generated by *Xho* I / *Cla* I restriction digest or by PCR using primers pBSK-zeo 5’ (GTAATACGACTCACTATAGGGCG) and pBSK-zeo 3’ (AAGCGCGCAATTAACCCTCAC) and purified from agarose gel using a Wizard column (Promega). DNA fragments were chromatinized using purified HeLa core histones, and a NaCl gradient dialysis protocol [55, 56]. Different ratios of histone to DNA (μg/μg) were used to produce different levels of nucleosome coverage. The ratios used in this study were low ratio (0.37/1, calculated to give two nucleosomes on 1.2 kb), medium ratio (0.74/1, calculated to give four nucleosomes on 1.2 kb) and high ratio (1.3/1, calculated to be in excess of histones for the maximum nucleosome coverage of 1.2 kb).

### Atomic Force Microscopy

For generation of Atomic Force Microscopy (AFM images), freshly cleaved 9.9 mm mica discs (Neyco S.A., Paris) were coated with 1 mM spermidine for five minutes, washed three times with water and dried with argon gas. Five ng of polynucleosome template diluted in 20 μl TE low buffer was deposited on the mica for two minutes, washed once with water and dried with argon gas. AFM was performed with a Nanoscope IIIa microscope (Digital Instruments, NY, USA) equipped with a type-E scanner and Nanoscope V controller (Bruker, CA, USA). AFM images were taken in tapping mode, using high-resolution silicon probes (RTESPA by Bruker, CA, USA). 1 × 1 μm images were recorded at a resolution of 512 × 512 pixels. The raw AFM images were processed with Nanoscope software.

### MNase digestion of reconstituted chromatin

Reconstituted chromatin was digested with 0.008 U/mL of MNase, 20mM of NaCl and 30 mM CaCl_2_, for 3 min at 28°C. This MNase concentration was selected from a concentration gradient tested to produce a mononucleosome band without overdigestion. Reactions were stopped by adding EDTA to a final concentration of 20 mM. Samples were then treated with 1 μl PNK enzyme (New England Biolabs) for 1 hour at 37°C, and digested DNA was separated on agarose gel. The band corresponding to the mononucleosome was excised from the gel. For the DNA alone control, double quantity of DNA was digested compared to the polynucleosome sample, and from the resulting DNA smear a fraction migrating between 100–300 bp was excised from the gel. DNA was purified on a Wizard column (Promega).

### *In vitro* Integration Assays

The IN-LEDGF/p75 protein complex was a gift from Marc Ruff, IGBMC, Strasbourg [20]. IN enzyme and LEDGF/p75 cofactor were purified as previously described [37, 57]. Two integration protocols were tested. The first protocol was adapted from [58] with minor changes. Briefly, reactions were conducted in 20–50ul reaction volume containing 100 mM NaCl, 20 mM Hepes pH 7.4, 12% DMSO, 10 mM DTT, 10 mM MgCl_2_, 20 μM ZnCl_2_. 10 nM of SupF pre-processed donor (generated by *Nde* I enzyme digestion) was added to IN alone (equivalent 600 nM monomer) or to the IN-LEDGF/p75 complex (equivalent 200 nM monomer) and incubated on ice for 30 min. 4 nM of acceptor DNA was added for a further 30 min on ice, then the reaction was shifted to 37°C for 1 hour. Reaction was stopped by the addition of 0.1% SDS, 1 mg/ml BSA, 10 mM EDTA and 1 μg/μl PNK enzyme. Integration products were then purified on a Wizard column (Promega). The second integration protocol has been previously described [27].

### PCR of integration products

The 5’ and 3’ pBSK-zeo primers, and U3 (TGGAAGGGCTAATTCACTTAACG) and U5 (ccgctgtggaaaatctctagca) primers targeting SupF were used to amplify integration sites. An alternative U3 primer (cggtcgcgcaattctttcggac) was selected for the DX014 sequence to avoid non-specific priming. Integration products were used as a template in 20 μl PCR reaction using 4 primer combinations (5’ pBSK-zeo/U5, 5’ pBSK-zeo/U3, 3’ pBSK-zeo/U5, 3’ pBSK-zeo/U3). PCR products were pooled and purified on a Wizard column (Promega).

### Sequencing and data analysis of MNase digestion products and integration products

DNA libraries consisting of either MNase digestion products or integration products obtained under different conditions were generated. The libraries were fragmented on a COVARIS S220 Focused-ultrasonicator using manufacturer recommendations to achieve a 350 bp mean fragment size. The libraries were constructed using a 'SPRIworks System I for Illumina Genome Analyzer' from Beckman and Illumina adapters from the 'TruSeq DNA Sample Preparation kit'. The resulting ligated fragments were PCR amplified and size selected on agarose gel. 74 bp paired-end sequencing was performed on an Illumina Genome analyser IIx (IMAGIF platform, Centre de Génétique Moléculaire, Gif-sur-Yvette, France). Integration sites were taken at the junction between the target DNA sequence and U5 or U3 viral ends. The nucleosome positions (V-plots) were obtained by plotting the lengths of MNase digestion products versus the position of their midpoints along the target DNA sequence [59]. From this V-plots, we derived the corresponding experimental occupancy landscape P(s), ie the total coverage in MNase-digested DNA fragment at the position s: starting from P(s) = 0,s = 1…L, for each point i of the V-plot (Xi = position of the middle of the fragment, Yi = the size of the fragment) we increment the occupancy value P at position s if this position is covered by the corresponding fragment: s in [Xi-Yi/2,Xi+Yi/2]. P was then normalized P-> P/$\sum_{1}^{L}P$.

### Prediction of nucleosome occupancy

When focusing on the dynamical assembly of histone octamers along the DNA chain, chromatin can be reasonably modelled by a fluid of 1D rods of finite extension l (the DNA wrapping length around the octamer), binding and moving in an external potential E(s) (the effective nucleosome formation potential at genomic position s) and interacting through a hard core potential of size l. Within the grand canonical formalism, considering that the fluid is in contact with a thermal bath (at temperature T) and a histone octamer reservoir (at chemical potential μ), the equilibrium density *ρ*(*s*) of hard rods in an external field E(s) obeys the nonlinear integral equation derived by Percus [54, 60]. From this equation, given E(s), μ and l, we numerically compute *ρ*(*s*) using the Vanderlick integration scheme [54, 61–63]. From the local density ρ(s) (ie the probability of having a nucleosome at the position s) we then can compute the occupancy landscape P(s) (i.e. the probability of a given site s to be occupied by a nucleosome) by the following convolution: P(s) = (ρ ∘∏_146_)(s) where ∏_146_(s) is defined by: ∏_146_(s)(s) = 1, s ϵ [–73,73] and = 0 elsewhere.

The mean density, ie the mean number of nucleosome (<N>) on a DNA fragment of total length L, is simply given by: <N> = $\sum_{1}^{L}\rho \left(s\right)$; when increasing μ, <N> increases with a titration curve <N> vs μ that depends on E(s) and thus, here, on the DNA sequence. Both ρ(s) and its coarse-grained version P(s) characterise the positioning of nucleosome along the sequence. For the parameter, we chose l = 146 bp which correspond to the average wrapping length around an octamer. The energy profile E(s) corresponds to the elastical energy computed as explained in [54] using a window size of 125 bp. We have actually renormalized this energy so that typical fluctuation of the resulting energy profile is 2 kT.

### Prediction of IN binding sites from DNA deformation energy

We predicted the IN binding preferences, based on the propensity of the DNA sequence to accommodate the strong mechanical deformations in the IN/LEDGF/DNA complex. The employed DNA mechanical energy of a 31 bp window was estimated from the base-pair step deformations, in the structural model proposed in [20]. The DNA sequence-dependent elastic parameters were derived from the conformational analysis of an extensive crystallographic database [64]. Using these parameters, the analysed sequences obtained from *in vitro* (this study) or *in vivo* experiments [21, 22] were threaded on the DNA shape within the complex. The resulting energy profiles E_IN_(s) exhibit important fluctuations, which are related to the experimental noise in the analysed structures: they were rescaled so that the standard deviation of the resulting profile is in the range of 2 kT. The integration preferences were then obtained from the Boltzmann weight at the different sites: ρ_IN_(s) = exp(-E_IN_(s)/kT).

## Results

### Selection and characterization of chromatin templates for *in vitro* integration assays

**Fig 1:**
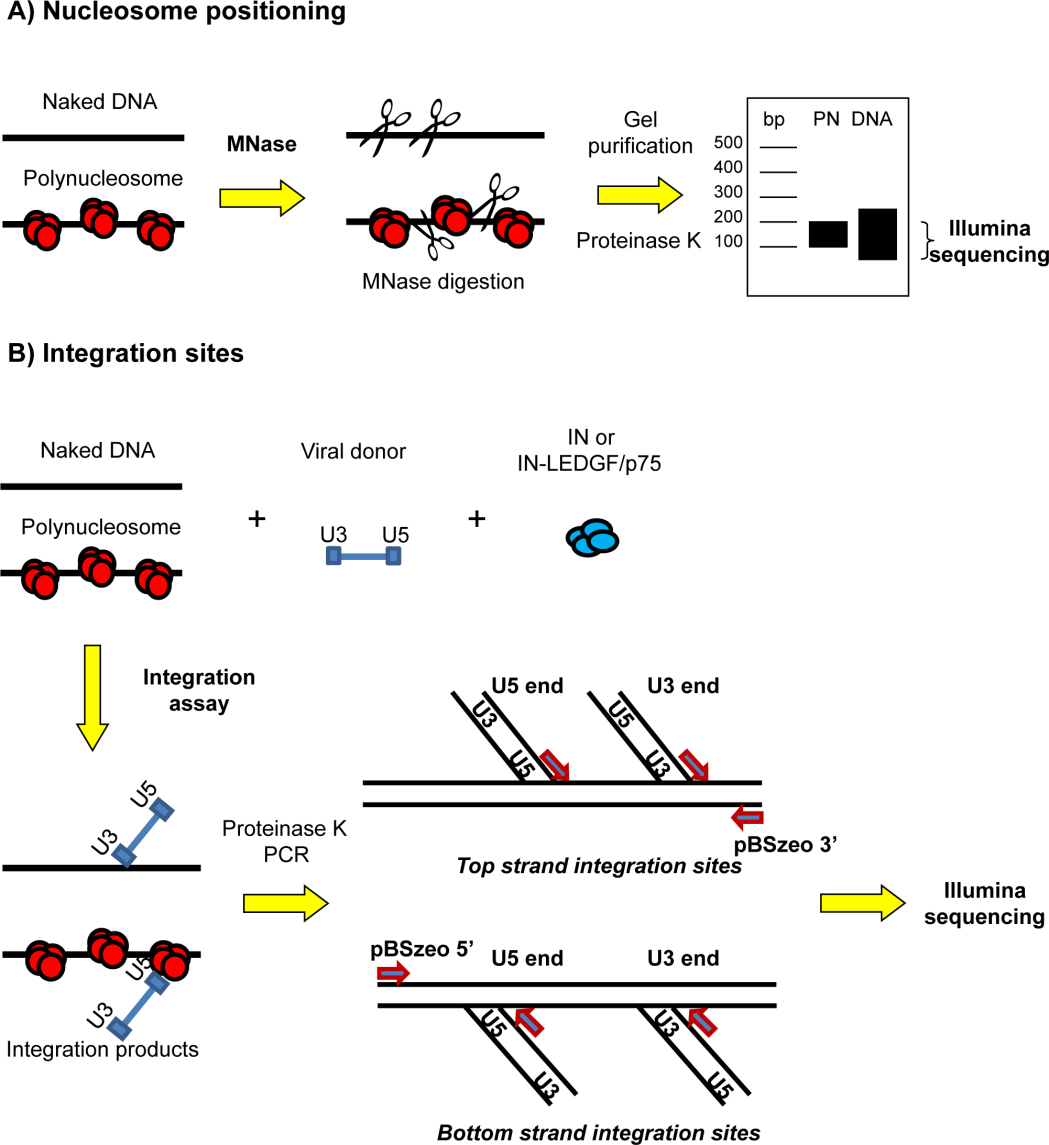
Schematic diagram of the experimental strategy on the selected sequences. A) Nucleosome positioning. To obtain nucleosome positions, naked DNA control, or *in vitro* assembled PN templates were digested with the Miccrococal nuclease (MNase) at a concentration optimal for obtaining a discrete mononucleosome band, and the bands were cut and extracted from an agarose gel and deep sequenced using Illumina technology. B) Integration site mapping. To obtain integration sites on the same DNA or polynucleosome templates, *in vitro* integration assays were performed with SupF viral donor and purified recombinant IN enzyme, or IN co-purified with LEDGF/p75. Integration products were deproteinized by proteinase K treatment, then used as templates for a PCR with primers specific to the U3 and U5 viral DNA ends, and primers common to the 5’ or 3’ ends of the DNA fragment. PCR products were pooled and deep sequenced using Illumina technology. Top strand integrations give a forward read PCR product and bottom strand integrations give a reverse read.

The aim of this study was to compare nucleosome positions and integration sites on chromatinized human DNA fragments containing an integration site identified *in vivo*. Our hypothesis was that polynucleosomes formed on natural DNA sequences would provide new information on the parameters of HIV-1 IN selectivity. The strategy of our *in vitro* experimental approach is summarized in Fig 1. On human DNA sequences containing an HIV-1 integration site identified in infected cells, we chose to study both nucleosome positioning using the MNase-seq strategy (Fig 1A) and integration on naked (unchromatinized) or chromatinized linear templates derived from these sequences (Fig 1B). The precise protocols will be described in more details in the following sections.

**Fig 2:**
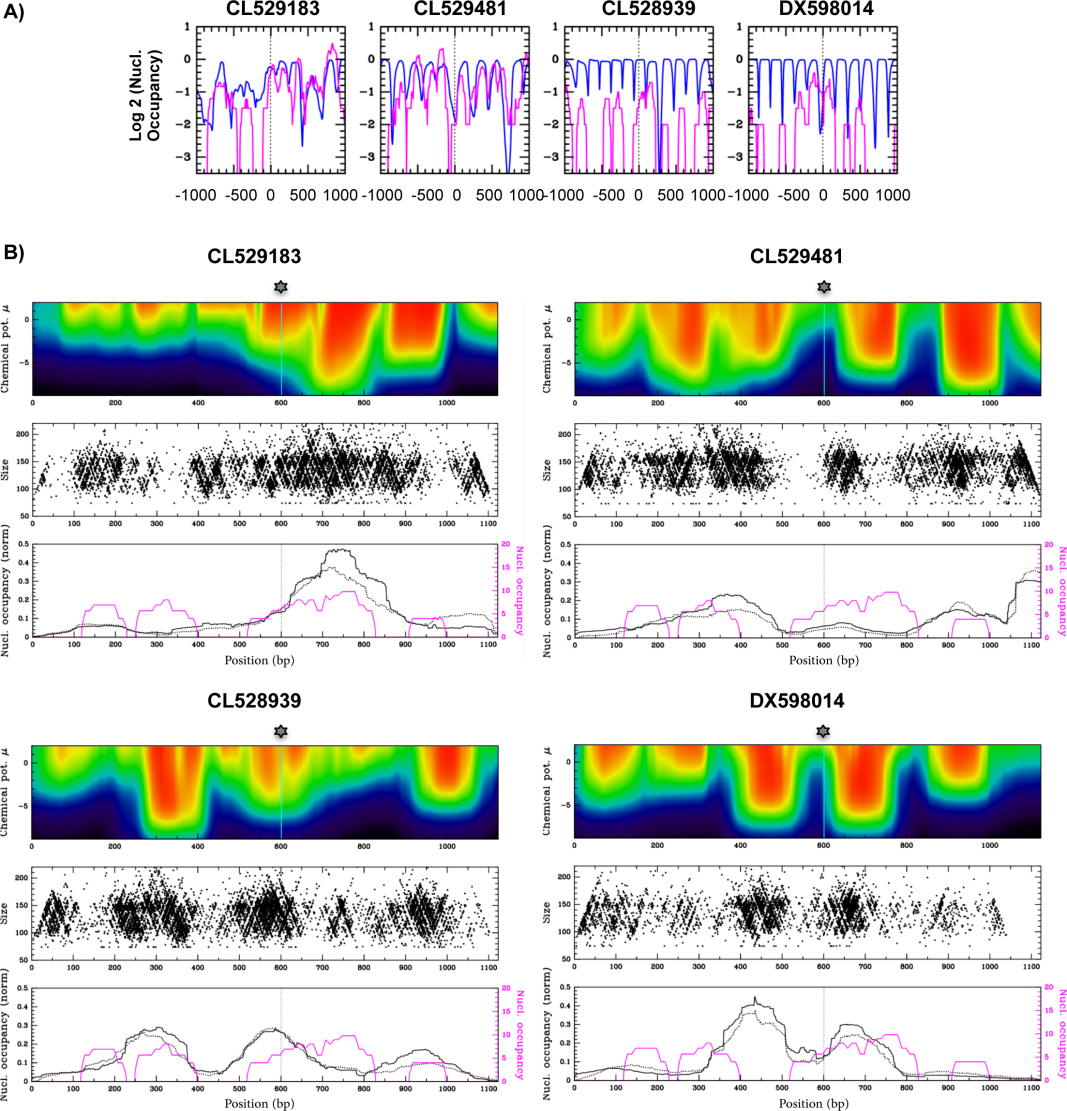
Nucleosome positions along four selected sequences. A) Predicted [66] (blue line) or experimentally derived [52] (magenta line) nucleosome occupancies (log2 values) around four HIV integration sites identified in infected cells: CL529183, CL529481 and CL528939 [11] and DX598014 [13]. Analysis are presented along 2 000 bp windows centred at these sites. B) PNs were assembled *in vitro* on 1.2 kb DNA fragments corresponding to the four selected sequences and centred on the position of *in vivo* identified integration site. Nucleosome positions were either predicted (upper panels) or mapped by MNase seq (middle and lower panels). Upper panels: heat maps of predicted nucleosome occupancies P(s) (defined in Materials and Methods). These occupancies were calculated along the studied sequences (positions on X axis) as a function of the chemical potential μ (Y axis) using an algorithm described in [61]. On these maps, dark blue corresponds to low probability and red to high probability. Middle panels: MNase digestion products of PNs assembled at one histone/DNA ratio (0.74 μg/1 μg) are represented by black points along the four sequences, according to their centre (X axis) and size (Y axis). To clarify this representation, only one tenth of the total MNase seq products are plotted. Lower panels: Nucleosome occupancies calculated from the MNase digestion products of PN assembled at two histone/DNA ratios (0.74 μg/1 μg, black solid line; 1 μg/1 μg, black dot line) (nucleosome occupancy values at a given site correspond to the total number of paired-end reads of MNase digestion products that covers this site, see Materials and Methods for more details). On the same panel is represented the nucleosome occupancy calculated from MNase-seq of cellular chromatin [52] (magenta line).

We first selected 1531 integration sites identified in different cell types [10–13] and compared the predicted and *in vivo* nucleosome positions [52] around each integration site. It should be noted that recent studies have since provided a vast number of HIV integration sites identified *in vivo* (for a recent study see [65]), but we chose not to increase the number of sites included for this particular analysis. Both the prediction and the experimental data represent steady state nucleosome occupancy and we can only postulate from these profiles that a nucleosome was present or not, at the time of integration. Across the selected integration sites, we observed a high diversity of nucleosome positions profiles, and selected four DNA sequences representative of this diversity (Fig 2A). Integration sites identified in sequences CL529183 [11] and CL528939 [11] are located within a nucleosome whereas the sites in sequences CL529481 [11] and DX598014 [10] are located in a linker region. Sequences CL529183 and CL529481 display irregular nucleosome positioning profiles, whereas sequences CL528939 and DX598014 are characterized by a more regular distribution.

Approximately 1.2 kb of these four sequences, centred around the *in vivo* HIV integration site were cloned, PCR amplified and the corresponding DNA fragments were assembled into chromatin at different histone/DNA (μg/μg) ratios. NaCl gradient dialysis and native HeLa histones were chosen for the chromatin assembly protocol, a system which favours thermodynamic nucleosome positioning [55, 56] and has already been used in *in vitro* studies on the 5S sequence [27, 28]. We used Atomic Force Microscopy (AFM) to count the number of nucleosomes present on each template assembled at different ratios (S1 Fig, panel A for sequence DX598014 at one assembly ratio). As expected, this number increased with larger histone/DNA ratios. As an example, polynucleosomes (PNs) assembled at four different histone/DNA ratios on the DX598014 sequence showed an increased nucleosome occupancy between ratios of 0.37/1 and 1.07/1 and a saturation above this ratio, corresponding to one nucleosome every 255 bp and an average linker of 110 bp (S1 Fig, panel B, and data not shown for other sequences). Nucleosome occupancy also differed between the selected sequences. At a given assembly ratio (for example 0.74 μg histone for 1 μg DNA), a higher average number of nucleosomes was obtained for some DNA sequences compared to others (3.03 for DX598014 compared to 2.44 for CL529183, S1 Fig panel C), indicating that some sequences are more favourable for nucleosome assembly. We observed similar differences when the number of nucleosomes covering each sequence was predicted with different values of the chemical potential μ (S1 Fig, panel D). In conclusion, nucleosome occupancies measured by AFM on the four selected PN templates depend on both histone/DNA ratio and on intrinsic DNA properties and the resulting templates are physiologically relevant for further integration studies.

We next used MNase digestion and paired-end sequencing (MNase-seq) to identify the precise nucleosome positions on our PN templates (strategy presented in Fig 1A). Digested PN products displayed an average length of 148 bp, consistent with the length of DNA wrapped around a mononucleosome (146 bp) whereas naked DNA digestion products were between 100–300 bp in length, corresponding to the size of fragments cut from agarose gels. Paired-end sequencing and nucleotidic alignments of the MNase digestion products allowed us to position them along the original sequences. Each digestion fragment was plotted according to its dyad position (midpoint of the digestion products) along the x-axis, and its length on the right y-axis (Fig 2B, middle panels, blue dots). This procedure resulted in a V-plot representation of the nucleosomes similar to that previously described by [59]. For each DNA sequence, 200,000–700,000 reads were analysed. Nucleosome occupancies were calculated from these V-plots (see Materials and Methods for the calculation) and represented along the four sequences (Fig 2B, lower panels, black curves, solid and dot lines). These *in vitro* nucleosome occupancies were compared with nucleosome positions predicted at different nucleosome densities [54] and represented by a heat map (Fig 2B, upper panels). The nucleosome occupancy profiles determined along these four sequences in CD4-T cells [52] are also represented on this figure (Fig 2B, lower panels, magenta curves).

This approach was first performed on PN templates assembled with a low assembly ratio (0.74 μg histone/1 μg DNA) and thus a low nucleosome coverage. The V-plots, and even more strikingly the nucleosome occupancy profiles calculated from these plots (Fig 2B, middle and lower panels), clearly indicated that the majority of nucleosome positions correlate very well with the predicted positions (Fig 2B, upper panels). This result was expected since both *in vitro* thermodynamics and *in silico* predictions of nucleosome positioning primarily depend on the DNA-sequence. Conversely, nucleosome occupancy profiles identified in cells (Fig 2B, magenta curve, lower panels) only partially correlated with *in silico* and *in vitro* profiles, consistent with the fact that DNA sequence is not the only determinant of nucleosome positioning within cells. This MNase-seq approach was also performed on the naked DNA templates and we compared the MNase digestion products obtained on naked and chromatinized templates (V-plots presented in S2 Fig). This comparison shows that the DNA sequence specificity of MNase is not responsible for the digestion profiles obtained on the chromatinized templates.

We also assessed whether varying the density of nucleosomes would change their positions. PNs were assembled at a higher ratio (1 μg histone/1 μg DNA) and MNase digestion products were used to calculate the nucleosome occupancies at the two different histone/DNA ratios along the four sequences (Fig 2B, lower panels, compare solid and dot lines). This study revealed strikingly similar nucleosome positions at both ratios. The *in vitro* nucleosome positions on these sequences are thus stable across different chromatin densities. Note that the naked DNA control digestion profile was distinctly different from the MNase positions on PN substrates (data not shown), revealing no significant cleavage bias, consistent with other reports of MNase usage on assembled chromatin [67]. In conclusion, the nucleosome positions identified by the MNase seq approach were a valid characterization that could be used for further *in vitro* integration studies.

### Efficiency of integration in naked and chromatinized templates

We first tested different protocols and IN preparations to obtain the optimal conditions for an *in vitro* study of IN efficiency and selectivity, in the absence and presence of LEDGF/p75. We used a 250 bp viral donor substrate containing the SupF gene and flanked by the pre-processed U3 and U5 ends [68]. IN (prepared in *E coli* according to [57]) was added to the reaction either alone or in the presence of LEDGF/p75. Since the chronology of addition of LEDGF/p75 with regards to the IN-viral DNA complex formation could interfere with the IN activity, we tested two different procedures of LEDGF/p75 addition. We first used a functional IN-LEDGF/p75 complex that has been shown to be more active than IN alone in both one end and two ends concerted integration reactions [43]. We also tested the addition of LEDGF/p75 to a preformed IN/donor DNA complex, this chronology favouring LEDGF/p75-dependent activation of integration into chromatinized templates [27]. Finally, we focused our study on HSI products (for both efficiency and selectivity studies), since under our selected experimental conditions these products represent the large majority of obtained integration products.

**Fig 3:**
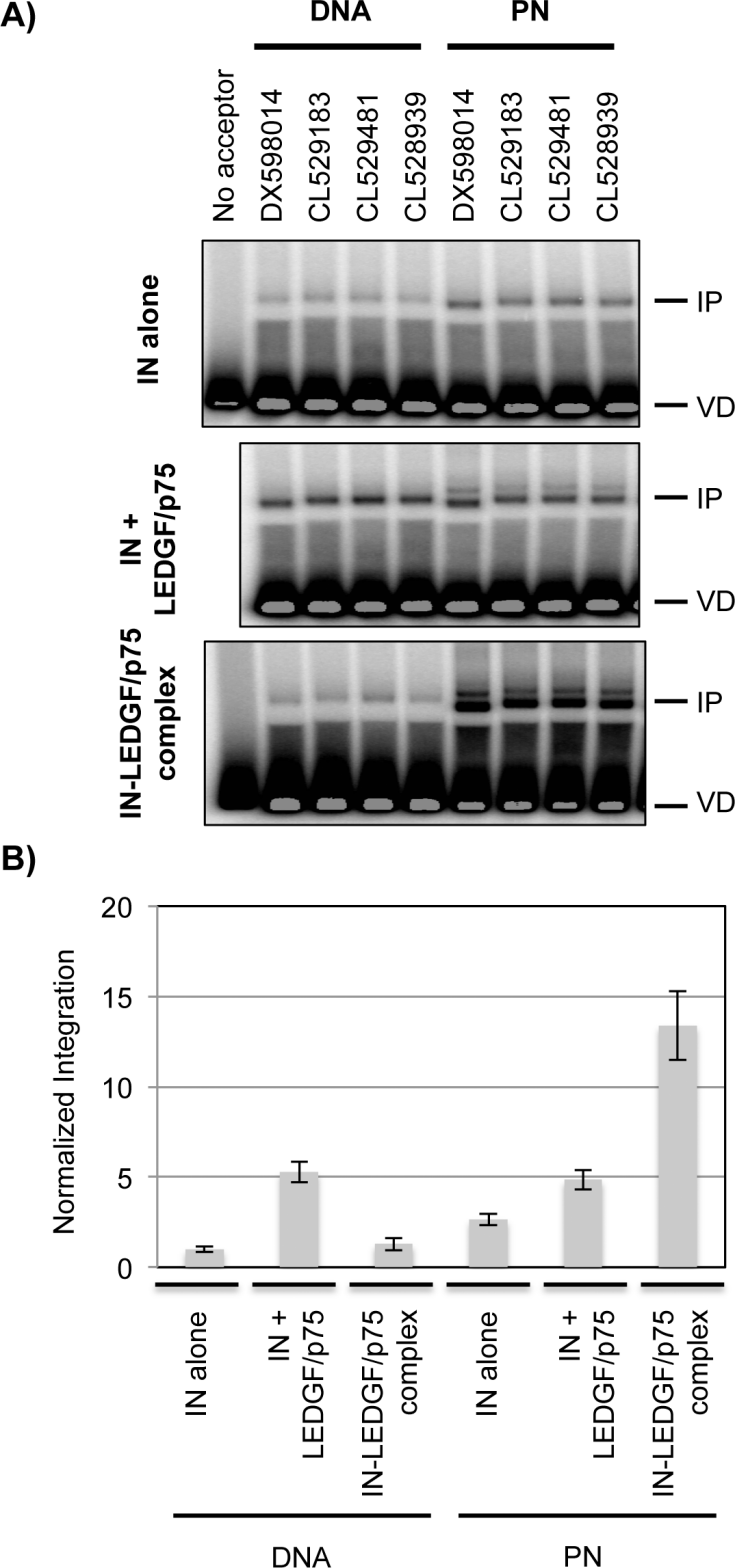
*In vitro* integration into selected naked and chromatinized templates. A) 1.2 kb DNA fragments of selected sequences (DX598014, CL529183, CL529481, CL528939) and PN assembled on these fragments at 1.3 μg/1 μg histone/DNA ratio, were used as integration acceptor templates. Integration assays were performed *in vitro* using a protocol adapted from [27], a radiolabelled U3-SupF-U5 donor and either IN alone, IN complemented by LEDGF/p75 after the formation of the IN-viral DNA complex [27] or the IN-LEDGF/p75 preformed complex [20]. Integration products were deproteinized, separated on a 1.2% agarose gel and revealed with a Fuji radioactivity imager. IP and VD correspond to the Integration Products and Viral Donor. B) Integration products were quantified under the different conditions, averaged for the four sequences and normalized for the average value obtained with IN alone in the DNA acceptor templates.

Using a radiolabeled viral donor and the protocol derived from [27], we evaluated the integration efficiency into the four selected templates, either naked or chromatinized by nucleosome assembly at a histone/DNA ratio of 1.3 μg/1 μg (Fig 3). Several observations can be made from this study. First, both IN alone and the IN-LEDGF/p75 complex are more active for integration into PN than into naked DNA and this difference is greater with IN-LEDGF/p75 (average > 10 fold) than IN (average 2.7 fold). This result was obtained on the four selected templates but also on the previously used 2.6 kb templates containing repeats of 5S nucleosome positioning sequences [27] (data not shown). This differential was not observed when LEDGF/p75 was added to a preformed IN-viral DNA complex, which differs from results previously obtained with different donor and acceptor substrates [27]. We propose that the length of the viral DNA substrate could be responsible for this difference. However, we clearly reproduced the LEDGF/p75-dependent activation of integration into the PN templates [27] and observed that this activation was more important with the IN-LEDGF/p75 preformed complex (5 fold) compared to the addition of LEDGF/p75 to a preformed IN-viral DNA complex (1.8 fold average activation) (Fig 3B). Finally, we optimized the integration reactions into PN templates with the IN and IN-LEDGF/p75 enzymes and compared two different protocols of integration adapted from [58] or [27] (S3 Fig). With both protocols, the IN-LEDGF/p75 complex was always more active than IN alone. Integration was more efficient using the protocol adapted from [58] (in S3 Fig, both gels were exposed for the same time) although it generated more integration products, which probably correspond to multiple integration of the radiolabeled donor substrate in the acceptor template. The optimal integration efficiency was therefore obtained using the IN-LEDGF/p75 complex, a PN acceptor template and a protocol adapted from [58]. These conditions were preferentially selected for our study on the effect of nucleosomes on IN selectivity.

### Selectivity of integration in naked and chromatinized templates

**Fig 4:**
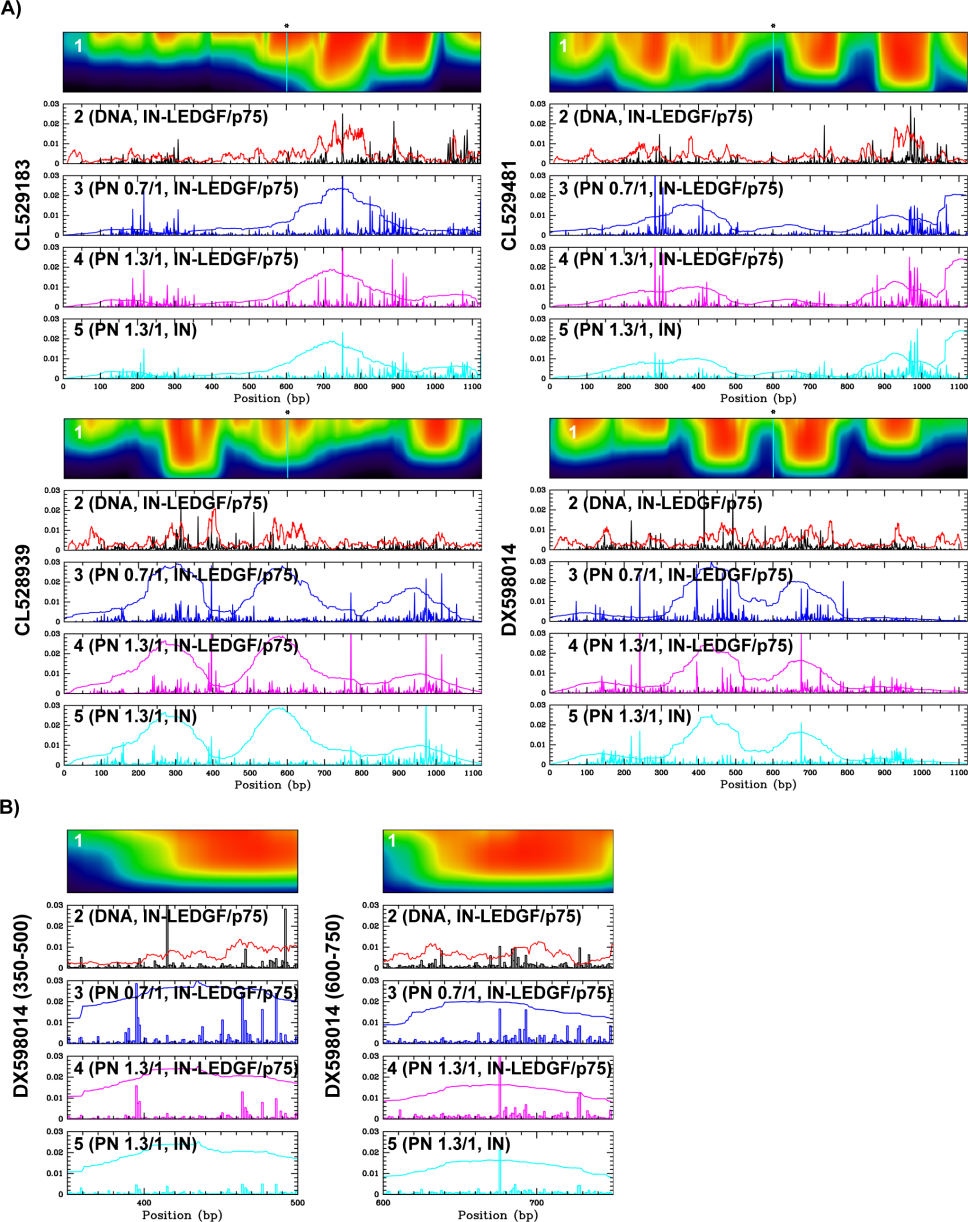
Integration sites and nucleosome positions along the four selected sequences. A) Integration sites identified *in vitro* on the four selected sequences and compared to nucleosome positions. Panels 1: Heat maps of nucleosome occupancy predicted at various nucleosome densities along the four selected sequences (CL529183, CL529481, CL528939 and DX598014). Panels 2 to 5: Integration sites identified on the four selected templates under different conditions: IN-LEDGF/p75 on DNA (panels 2), IN-LEDGF/p75 on PN assembled at histone/DNA ratio 0.74 μg/1 μg (panels 3) or 1.3 μg /1 μg (panels 4) and IN alone on PN assembled at histone/DNA ratio 1.3 μg /1 μg (panels 5). Integration event reads at each position were normalized to total integration event read numbers. Integration sites were compared to predicted IN binding preference based on DNA physical properties (red curves panels 2) (ρ_IN_(s)) or to nucleosome positions obtained by MNase-seq (blue, magenta and cyan curves, panels 3 to 5). Experimental conditions of integration corresponding to panels 2 to 5 (respectively libraries L6 to L9, respectively) are summarized in Table 1. B) Integration sites identified *in vitro* on two nucleosome-covered regions of the DX598014 sequence. Panels 1 to 5: similar analysis as in Fig 4A restricted to nucleotides 350–500 and 600–750.

Our goal was to determine whether *in vitro*, HIV-1 integration preferentially occurs in nucleosome occupied region, and whether LEDGF/p75 regulates this selectivity. Given the results obtained regarding integration efficiency (Fig 3), we started this study using the IN-LEDGF/p75 complex, a protocol adapted from [58], and DNA or PN templates assembled at two different histone/DNA ratios. The integration products were amplified by PCR with primers targeting the 5’ and 3’ ends of the acceptor DNA and the U3 and U5 viral DNA ends in the SupF donor (strategy presented in Fig 1B). This PCR cannot detect donor-donor integration products but only donor-acceptor products. It can distinguish between integration products in the top and bottom strands, as well as integration from U3 or U5 ends. PCR products were pooled into libraries corresponding to different experimental conditions, sequenced and aligned against the sequences of the four selected templates. Alignments gave the precise sites of integration. Experimental conditions corresponding to each integration sites libraries are summarized in Table 1 and corresponding statistics are listed in S1 Table. Fig 4 presents the position of integration sites determined along the four selected sequences, under various selected experimental conditions.

**Table 1:** Experimental conditions corresponding to the different *in vitro* integration sites libraries.

Library	Template	Integrase	PCR cycles
L6	DNA	IN-LEDGF/p75	35
L7	PN ratio 0.7/1	IN-LEDGF/p75	35
L8	PN, ratio 1.3/1	IN-LEDGF/p75	35
L9	PN, ratio 1.3/1	IN	35
L12	DNA	IN-LEDGF/p75	15
L13	PN, ratio 1.3/1	IN-LEDGF/p75	15

Integration sites were obtained with IN-LEDGF/p75 on DNA (L6, L12), IN-LEDGF/p75 on PN assembled at histone/DNA ratio (0.74 μg/1 μg) (L7), IN-LEDGF/p75 on PN assembled at histone/DNA ratio (1.3 μg/1 μg) (L8 and L13), IN on PN assembled at histone/DNA ratio (1.3 μg/1 μg) (L9). L6 to L9 and L12/L13 correspond to sites identified using two different PCR amplification protocols.

On naked DNA, integration sites seem to be enriched in the nucleosome occupied regions, even if the nucleosomes are not present on the templates (Fig 4, panels 2). This result could reflect the fact that both nucleosome positioning and IN binding require similar structural deformations of the DNA. Concerning IN binding, a strong distortion of the acceptor DNA has been reported in the EM structure of the HIV-1 IN-LEDGF/p75-DNA complex [20]. Using a DNA elastic model based on crystallographic data [64], we estimated the sequence-dependent mechanical cost associated with this deformation which allowed us to predict the IN binding preferences along the four selected sequences (for more details see Materials and Methods). This computed probability of IN binding (Fig 4, panels 2, red curve) was compared to integration sites identified on naked DNA along the four studied sequences (Fig 4, panels 2, black bars) and a weak correlation was observed between them (Pearson correlation coefficient between 0.2 and 0.6, S2 Table). Therefore, the DNA physical properties used to calculate the IN binding preferences could partially explain the choice of integration sites *in vitro* into naked DNA. This role will be tested at a genomic level on integration sites identified in cells (see the last section of results).

Integration sites of the IN-LEDGF/p75 complex were then mapped in the PN templates assembled on the four selected sequences at two histone/DNA ratios (0.74/1 and 1.3/1) (Fig 4, panels 3 and 4, blue and magenta bars) and compared to nucleosome positions. For this comparison, we used both predicted nucleosome positions (Fig 4, panel 1, heat maps) and *in vitro* nucleosome positions derived from MNase seq data (Fig 4, panels 3 and 4, blue and magenta curves). An enrichment of integration sites was most often observed in regions corresponding to high nucleosome occupancies (nucleotides 600–950 in CL529183, 150–500 and 850–1050 in CL529481, 250–450 and 900–1100 in CL528939 and 350–500 and 600–750 in DX598014, panels 3 to 5 of Fig 4A) and it was not globally affected by the nucleosome occupancy of the template (similar enrichment observed at two histone/DNA ratios). A detailed analysis of integration sites within the nucleosome covered sequences (for example, region 350–500 and 600–750 in DX598014, Fig 4B) revealed a better similarity between integration sites mapped into PN assembled at two different ratios than between integration sites mapped into DNA versus PN (compare panels 2 versus 3 and 4 of this figure). These observations suggest that chromatinization affects the precise distribution of integration sites within the nucleosome-covered regions. To quantify this effect, we performed correlation studies between integration sites identified in DNA versus PN (Table 2). Pearson correlation coefficients were calculated between integration sites identified in the four selected sequences under the three different conditions (DNA, PN assembled at low ratio and PN assembled at high ratio, corresponding to panels 2, 3 and 4 on Fig 4 and integration sites libraries L6, L7 and L8 in Table 1). Correlation values calculated between conditions DNA and PN low ratio or DNA and PN high ratio (between 0.29 and 0.58) are significantly lower than the correlation values calculated between conditions PN low ratio and PN high ratio (between 0.7 and 0.92). These values confirm that the distribution of integration sites in naked DNA significantly differs from the distributions in chromatinized templates.

**Table 2:** Correlation values between integration sites of the different librairies.

		L6	L7	L8	L9	L12	L13
CL529183	L6	1	0	0	0	0	0
	L7	0.60 ±0.04	1	0	0	0	0
	L8	0.34 ±0.10	0.87 ±0.03	1	0	0	0
	L9	0.60 ±0.05	0.86 ±0.02	0.78 ±0.02	1	0	0
	L12	0.85 ±0.02	0.64 ±0.03	0.40 ±0.04	0.69 ±0.02	1	0
	L13	0.42 ±0.06	0.78 ±0.03	0.79± 0.04	0.83 ±0.02	0.49 ±0.04	1
CL529481	L6	1	0	0	0		
	L7	0.55 ±0.08	1	0	0		
	L8	0.58 ±0.07	0.92 ±0.02	1	0		
	L9	0.78 ±0.03	0.75 ±0.03	0.85 ±0.02	1		
CL528939	L6	1	0	0	0	0	0
	L7	0.54 ±0.04	1	0	0	0	0
	L8	0.34 ±0.06	0.82 ±0.04	1	0	0	0
	L9	0.36 ±0.07	0.77 ±0.02	0.75 ±0.05	1	0	0
	L12	0.92 ±0.01	0.54 ±0.04	0.35 ±0.06	0.42 ±0.08	1	0
	L13	0.42 ±0.05	0.94 ±0.01	0.84 ±0.05	0.82 ±0.03	0.47 ±0.05	1
DX598014	L6	1	0	0	0		
	L7	0.29 ±0.04	1	0	0		
	L8	0.29 ±0.08	0.70 ±0.02	1	0		
	L9	0.44 ±0.06	0.48 ±0.05	0.77 ±0.03	1		

Pearson correlation values with standard deviations were calculated between integration sites identified *in vitro* on the four selected sequences (CL529183, CL529481, CL528939 and DX598014) and under the different experimental conditions reported in Table 1.

The numbers of PCR cycles and sequenced products could, however, affect the distribution of integration sites. To test the role of these parameters, we repeated the mapping of integration sites with a lower number of PCR cycles (15 instead of 35) and increased the number of sequences (between 100 000 and 500 000 reads instead of 3 000 to 100 000). This study was performed on naked or chromatinized templates of two sequences (CL529183 and CL528939) (integration sites libraries L12 and L13, Table 1). Correlation values were calculated between these libraries of integration sites and the previous libraries (Table 2). Again, high correlation values were measured between integration sites identified into naked DNA (0.85 to 0.92 for DNA 35 cycles versus 15 cycles, L6/L12) or into PN (0.79 to 0.84 for PN 35 cycles versus 15 cycles, L8/L13). Conversely, low correlation values were measured between integration sites identified in naked DNA versus PN (≈ 0.34 for DNA versus PN with 35 cycles, L6/L8 and ≈ 0.48 for DNA versus PN with 15 cycles, L12/L13). Therefore, neither PCR, nor sequencing steps are responsible for the changes of integration sites distributions.

We also repeated the analysis of integration sites on the four chromatinized templates using IN alone instead of the IN-LEDGF/p75 complex (Fig 4A, panel 5 and Table 1, Library L9). We observed a very good correlation between the integration sites obtained under both conditions (Pearson correlation values between 0.7 and 0.85, L8/L9 in Table 2). Therefore, at least for this *in vitro* experimental situation, LEDGF/p75 does not affect the distribution of integration sites into the PN templates. We can conclude from this study that the IN-LEDGF/p75 complex is sensitive to the chromatinisation of the acceptor template (Fig 3), as previously reported [27]. However, under our experimental conditions, LEDGF/p75 is not responsible for the changes of IN selectivity observed between DNA and PN templates (Fig 4 and Table 2). The targeting properties of LEDGF/p75 observed in infected cells probably require other reaction parameters absent in our assays or depend on another level of the chromatin organization in the nucleus (see discussion).

In summary, the high correlations observed between integration sites identified into naked DNA (libraries L6, L12) or between sites identified into PN (libraries L7, L8, L9 and L13) clearly demonstrate a chromatin-dependent selectivity of integration. A more precise analysis of the sites was then performed to determine if this difference is associated with a preferential integration in the nucleosomes.

### Quantitative analysis of integration sites in PN reveals a selectivity of IN for DNA structures induced by the presence of a nucleosome

**Fig 5:**
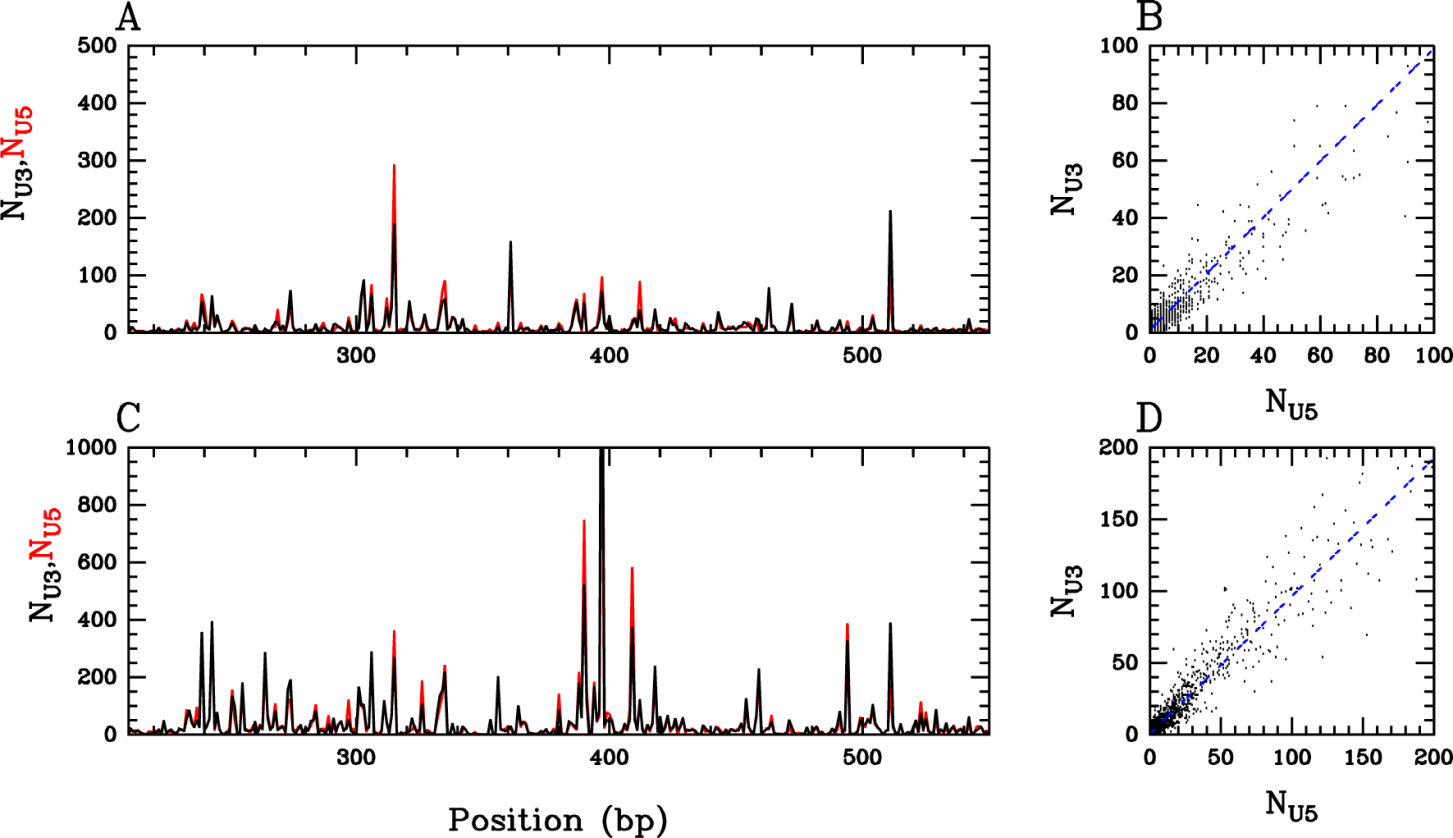
Comparison between U3 and U5 integration sites identified *in vitro* on sequence CL528939. This comparison is presented for integration sites identified with IN/LEDGF complex in naked (A and B) or chromatinized (C and D) template. U3 (black line) and U5 (red line) integration sites are either superposed along the sequence (A and C) or subject to correlation analysis (B and D).

Previous *in vitro* studies have shown that the U5 viral end integrates more efficiently than the U3 end, but haven’t explored the difference of integration selectivity by these two ends [69–72]. These data could help in understanding the integration mechanisms because *in vivo* integration sites correspond to a compromise between U3 and U5 integration selectivity. Our proposed protocol allows us to study this parameter because it favours half-site independent integration and can distinguish integration sites mapped from each end of the donor substrate. Comparing integration sites from U3 and U5 ends with the four templates, we observed a very good superposition of U3 and U5 integration sites on both DNA and PN templates (shown in Fig 5 for sites obtained into DNA (A, B) or PN (C, D) of the CL528939 sequence). This shows that the viral end is not involved in the integration selectivity.

**Fig 6:**
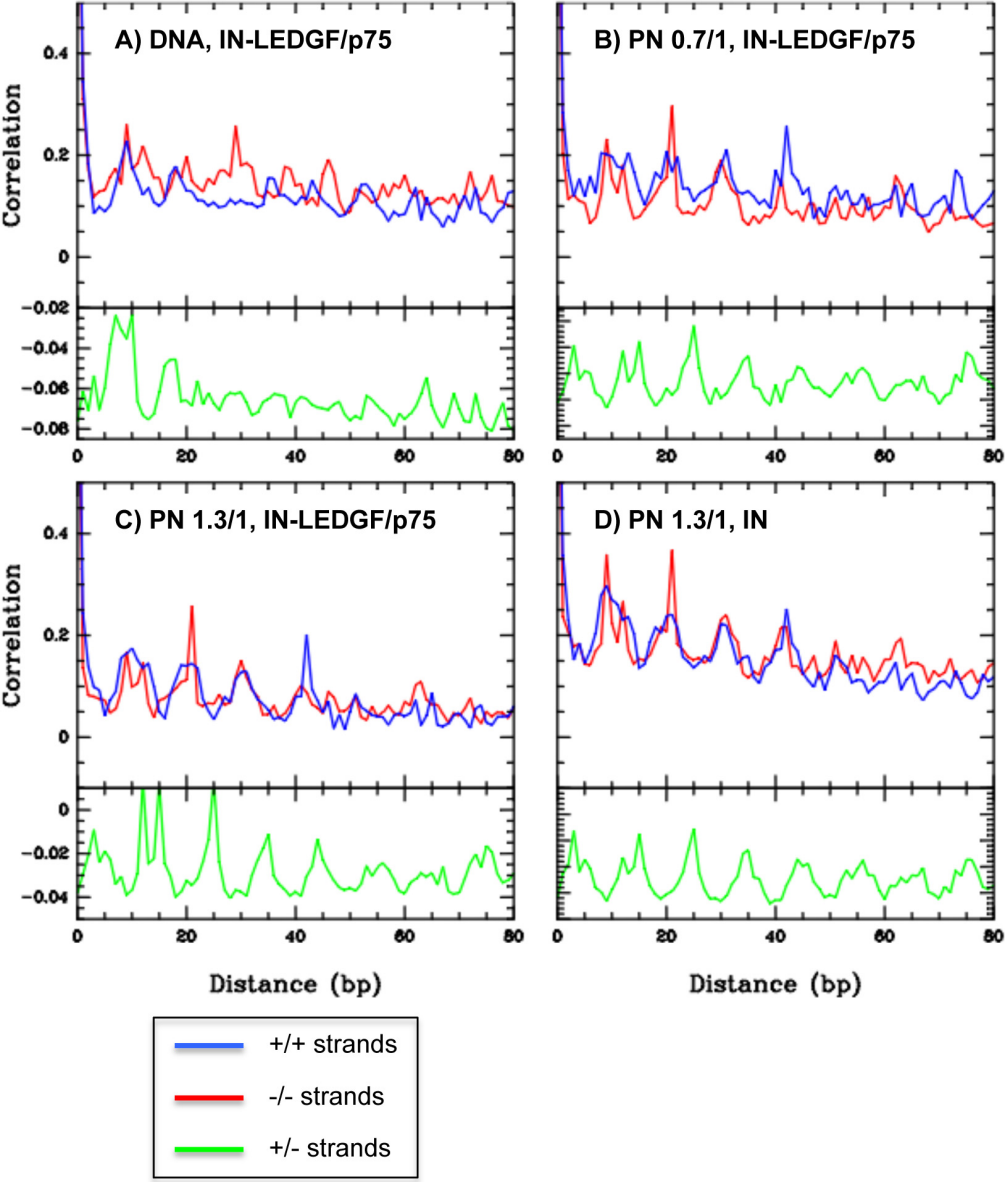
Autocorrelations between integration sites identified on three selected sequences (CL528939, CL529481 and CL529183). Autocorrelations were calculated between integration sites identified on three selected sequences and corresponding to different conditions of integration. A) IN-LEDGF/p75 on DNA, B) IN-LEDGF/p75 on PN assembled at histone/DNA ratio (0.74 μg/1 μg), C) IN-LEDGF/p75 on PN assembled at histone/DNA ratio (1.3 μg/1 μg) and D) IN on PN assembled at histone/DNA ratio (1.3 μg/1 μg). For each panel, autocorrelations were calculated between integration sites of the same strand (+/+ red and-/- blue) or complementary strands (+/- green).

Our assay also allows us to distinguish integration sites mapped on the top (+) and bottom (-) strands of the acceptor templates. This parameter is important since the autocorrelation curves between these sites indicate the orientation of the enzyme with regards to the acceptor template and also provide information on the structure of this template at the integration site. For this purpose, we sorted the integration sites obtained on the (+) and (-) strands with the IN-LEDGF/p75 complex and under three different conditions (DNA, PN low ratio and PN high ratio). These data correspond to a compilation of integration sites obtained on three templates (CL528939, CL529481 and CL529183) and already presented in Fig 4A (panels 2, 3 and 4) or analysed for their correlation in Table 1 (L6, L7 and L8). For these three different conditions, we first performed an autocorrelation between sites present on the same strand (Fig 6, blue and red lines are autocorrelation curves between sites on +/+ and-/- strands). With integration sites identified into PN templates (Fig 6, panels B, C and D), we observed a periodic peak of autocorrelation at 10, 20, 30 and 40 bp which suggests that the integration sites are located on the same side of the DNA helix, that likely corresponds to the outside of the nucleosome structure. This periodicity was not observed in the autocorrelation curves of integration sites identified into the naked DNA templates (Fig 6, panel A). This result suggests that periodic integration sites identified in the PN are independent of DNA sequence but depend on the presence of nucleosomes. Another parameter characteristic of HIV-1 integration is a 5 bp stagger between integration sites on the two strands, which corresponds to the target DNA major groove. We therefore performed an autocorrelation analysis between sites located on the (+) and (–) strands of the same sequences. The autocorrelation curves corresponding to sites identified into PN templates revealed a first peak of correlation located at 5 bp and following peaks every 10 bp (15, 25, 35 bp, etc…). This profile is consistent with an integration process targeting enlarged DNA major grooves facing out from the nucleosome. This repeated signal was not observed in the autocorrelation curves calculated between sites identified into naked DNA.

In summary, the autocorrelation curves obtained between integration sites mapped on the same or different strands of chromatinized templates demonstrate that the distribution of integration sites into these templates is not random. Periodicities observed in these curves are compatible with DNA structure and accessibility changes induced by a nucleosome and therefore support a preferential integration into nucleosome occupied sequences.

### Genome-wide analysis of DNA structure and nucleosome positioning around integration sites

We next carried out genome-wide analyses of the role of DNA structural properties and nucleosome positions as IN selectivity parameters.

**Fig 7:**
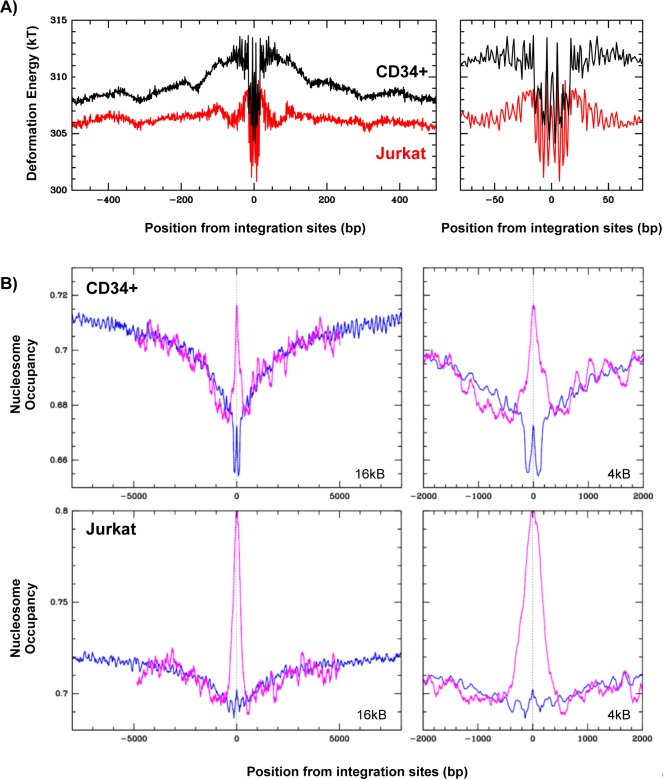
DNA deformation energy and nucleosome occupancy around HIV-1 integration sites. A) Compilation of the DNA deformation energy (see Materials and Methods) required to adopt the structure present in the IN-LEDGF/p75 intasome structure [20] calculated for a 31 bp window along genomic sequences surrounding integration sites identified in Jurkat [22] or CD34+ cells [21]. The compilations are presented along 1.2 kb (left panels) or 160 bp (right panels) windows centred around the integration sites. B) Genome wide compilations of predicted [66] (blue line) and experimentally derived nucleosome occupancies [52] (magenta line) around integration sites identified in CD34+ multipotent hematopoietic progenitor cells [21] (upper panel), and Jurkat cells [22] (lower panel). Compilations are presented along 16 kb (left panels) or 4 kb (right panels) windows centred around the integration sites.

First, since the target DNA helix is severely distorted by IN binding [19, 20], we hypothesized that the sequence-dependent mechanical cost of this deformation could be a key contributor to the integration free energy. Based on the propensity of the DNA helix to accommodate the deformation present in the IN-LEDGF-DNA structure [20], we calculated the energy profiles corresponding to IN binding along 1.2 kb sequences surrounding HIV-1 integration sites identified in Jurkat or CD34+ cells [21, 22], using a DNA elastic model based on crystallographic data [64] (for more details see Materials and Methods). The calculated energies were compiled and centred at the integration sites (Fig 7A, left panel). This study, similar to the one performed on the integration sites identified *in vitro* on naked DNA templates (Fig 4, panels 2), clearly revealed a global decrease of energy around the integration sites. This result obtained with two large sets of integration sites identified in infected cells suggests that the physical properties of DNA linked with IN binding can constitute a first level of IN selectivity. Interestingly, this decreased energy was associated with larger energy fluctuations in a 150 bp window that could correspond to a favourable nucleosome position (Fig 7A, right panel).

Nucleosomes have indeed been proposed to be a favoured target of HIV-1 integration in infected cells [15, 22], although we have also observed a preferential integration in genomic regions of weaker nucleosome density [28]. Given that these conclusions were obtained comparing integration sites in infected cells to predicted nucleosome positions [66, 73, 74], we decided to test whether similar correlations would be obtained with nucleosome positions identified *in vivo*. Such positions have been recently mapped in CD4-T cells using MNAse digestion of chromatin and high throughput sequencing of digested products (MNAse-seq) [52, 53]. We compared these nucleosome positions with two sets of HIV-1 integration sites identified in the T-lymphocyte Jurkat cell line [22] or in human CD34^+^ multipotent hematopoetic progenitor cells [21]. We also compared integration sites with predicted nucleosome positions according to [66]. Nucleosome occupancies determined *in vivo* were compiled around integration sites from these two libraries [21, 22]. The average occupancies were plotted along the sequences and centred at the integration site (Fig 7B for nucleosome maps from [52] and S4 Fig for nucleosome maps from [53], magenta lines). In both CD34^+^ and Jurkat cells, we observed a strong peak of nucleosome occupancy at the integration site and this peak was observed with both sets of nucleosome positions. We also observed a global decrease of the average *in vivo* nucleosome occupancy in the local area surrounding the integration sites (clearly visible in the 16 kb window) with the nucleosome map corresponding to activated CD4 T-cells [52] (Fig 7B) but not with the map corresponding to global CD4 T-cells [53] (S4 Fig). Similar results were obtained with predicted nucleosome occupancies (Fig 7B and S4 Fig, blue lines). Therefore, the comparison between *in vivo* nucleosome positions and integration sites shows a preferential integration of HIV-1 into nucleosomal DNA, supporting the previous conclusions derived from predicted nucleosome positions [15, 22, 28].

## Discussion

### A new *in vitro* integration assay in chromatin templates, improvements and limits

In this study, we developed a new *in vitro* integration assay with several major improvements. Firstly, we used acceptor chromatin templates assembled on natural human DNA sequences. Nucleosome positioning sequences used in previous studies [27–29, 51] affect the structural properties of the DNA helix and could perturb the integration process on naked DNA but also on the assembled nucleosomes, characterized by a higher stability [75]. We have already observed that stable and regularly spaced nucleosomes disfavour FSI and that SWI/SNF remodelling of these structures restores efficient integration [28]. Therefore, natural DNA sequences, naked or chromatinized, should provide more physiological integration acceptor templates to study the effect of both DNA and chromatin structure on the integration process. Furthermore, in the present study, nucleosomes were assembled by salt gradient dialysis that favours the lowest energy positions according to the DNA sequence. If the DNA sequence is not the only determinant of nucleosome positioning [76, 77], its effect on DNA-histone interactions may modulate the action of DNA-binding proteins and DNA-dependent enzymes. Recently, nucleosome positions around transcription promoters have been kinetically followed during viral infection (by Kaposi's sarcoma-associated herpesvirus) and have revealed a transient redistribution favouring DNA-directed nucleosome positions similar to the ones obtained using predictive algorithms or after *in vitro* salt-dialysis assemblies [78]. This study supports a mechanism in which the DNA sequence plays a role in nucleosome positioning, especially during cellular processes such as viral infection.

AFM coupled to MNase-seq. offers a major technical input to quantify and evaluate the nucleosome positions after *in vitro* assembly. AFM revealed significant differences between chromatin assembly efficiencies on the used sequences that correlated well with the predictions. MNase seq. is one of the most precise and less invasive tools to map nucleosomes or DNA binding protein positions along genomic sequences [59]. Using this approach, we did not observe any significant change of nucleosome positions on PN templates assembled at different histone/DNA ratios. Therefore, AFM coupled to MNase approach allowed us to evaluate the nucleosome density, spacing and stability on assembled templates before using them as integration acceptor substrates.

The third advantage of our integration assay is the generation and sequencing of a very large number of integration events on each acceptor template resulting in a high density of integration sites per bp of template. *In vivo* studies of integration sites cannot obtain such a density (for example in [22], a density of 40 000 sites is obtained for a 2.10^9^ bp genome, which corresponds to an average density of 1 site per 50 000 bp). *In vitro* studies of integration sites, performed by PCR with a radiolabeled primer [29], or by cloning non-radiolabeled integration products [68], give low densities of integration sites per bp that restricts their quantitative analysis, especially when they are compared to nucleosome positions. In contrast, the high density of integration sites per bp of template obtained with our new protocol is unique and allows a quantitative analysis of IN’s ability to repeatedly target the same site within a given sequence (Table 2 and Fig 6). For example, this high density enabled us to calculate autocorrelation factors between integration sites mapped either on same strands (+/+,-/-) or complementary strands (+/-) strands under each condition (Fig 5). While few peaks, and no apparent periodicities, were observed on naked DNA, autocorrelation curves calculated from integration sites mapped into the PN templates clearly showed peaks with a 10 bp periodicity (Fig 6, panels B, C and D). This periodicity is consistent with integration sites present on the same side of the DNA helix or induced by a regular DNA curvature, two parameters related to the nucleosome structure. Furthermore, the first peak of the autocorrelation curves calculated between complementary strands is located at 5 bp. This could be attributed to the 5 bp distance separating concerted integration sites, but more probably to preferential integration in the DNA major groove, enlarged by the nucleosome structure.

An initial observation from this study was the lack of enriched integration at the site identified *in vivo* in the selected sequences. This result, although disappointing, was not completely surprising and suggests that other parameters relative to the *in vivo* situation were lacking in our assay. There are several recent papers highlighting that chromatin organisation relative to the nuclear pore and nuclear envelope is important in integration site selection [65, 79]. Additionally, epigenetic signatures *in vivo* such as H3K36me3 probably direct LEDGF/p75-mediated targeting of integrase. In support of this notion, we did not observe any effect of LEDGF/p75 on integration sites selection in nucleosome-covered sequences (Fig 4 and Table 2). However, as expected, the interaction of LEDGF/p75 with IN stimulated its activity in the PN templates (Fig 3). These results obtained with LEDGF/p75 could have several explanations. Chromatin templates used in our study contain a global population of histone modifications and are not enriched in H3K36me3 that is known to interact with the LEDGF/p75 PWWP domain. To test this hypothesis, we introduced histones specifically modified with the H3K36me3 mark into several chromatin templates (as described in [80]) and tested the consequences on integration efficiency. Using several integration protocols (LEDGF/p75 added at different reaction times), we failed to observe any difference in the efficiency of integration with respect to acceptor chromatin templates containing the unmodified H3 histone (data not shown). These results do not rule out the role of the LEDGF/p75-H3K36me3 interaction as a parameter of integration, but suggest that this interaction plays a role in site selectivity rather than global efficiency. Our *in vitro* experimental conditions were optimized for the most efficient integration in PN templates and this optimization could disfavour selectivity. Even our choice of viral donor (SupF 250 bp) and length of viral acceptor could modify site selectivity, and longer sequences may lead to less efficient but more specifically targeted *in vitr*o integration. Other enzymatic conditions need to be explored in order to find those favouring a selective process. The LEDGF/p75-H3K36me3 interaction could also require additional cofactors, absent in our assay that could play a role during the integration process. We have recently identified two LEDGF/p75 PWWP partners, the TOX4 transcriptional activator and the NOVA1 splicing regulator, and the overexpression of their PWWP binding domain specifically inhibits HIV-1 replication [81]. These two proteins could play a role in the LEDGF/p75-dependent activation of integration into chromatin acceptor templates. Additionally, both MLL Trithorax and Bmi-1 Polycomb complexes functionally interact with LEDGF/p75 during transcriptional regulation and could also play a role during viral integration [82, 83].

Concomitantly with this study, we observed that retroviral INs (HIV-1, MLV and ASV) have different *in vitro* FSI selectivities in nucleosome-covered templates [51]. More precisely, in the case of HIV-1, *in vitro* FSI is favoured outside the nucleosome-covered sequences and this could be interpreted as conflicting with our data in the present manuscript. These different IN selectivities for nucleosomes probably result from different experimental conditions such as the density and stability of nucleosomes (assembled on natural versus repetitive nucleosome-positioning sequences), or the integration assay conditions (optimised for half site versus full site integration). In fact, these differences reveal new parameters of IN selectivity, such as the structural properties of the nucleosomes and the process of integration itself. In the future, comparing the structural constraints of both HSI and FSI processes for various nucleosome-covered sequences should be very informative on the mechanisms of retroviral integration. Furthermore, in both studies, we confirm our previous observation [28], that integration sites are preferentially located in nucleosomes surrounded by a low nucleosome density chromatin environment.

In summary, our new integration assay allows major technical improvements in quantitatively measuring the effects of DNA structure and/or nucleosomes assembled on non-positioning sequences on integration. Quantitative analyses of HSI sites obtained with this assay support a selective integration favouring nucleosome occupied sequences, even when they are assembled on non-positioning sequences. The selectivity of integration towards nucleosomes observed in the present study, correlates well with the enrichment of integration sites in nucleosome covered regions in infected cells *in vivo*, and this correlation is a strong endorsement of our experimental strategy. A major challenge will be to develop integration assays that take into account multiple selectivity parameters revealed from *in vivo* studies, such as histone modifications, and the transcriptional machinery, within the same assay.

### *In vivo* genomic studies reveal the link between two parameters of IN selectivity

DNA structural deformations are known to be determinant for the binding preferences of several proteins [84] or protein complexes such as nucleosomes [85]. HIV-1 IN probably belongs to this family of shape-readout DNA binding proteins as suggested by its preference for specific DNA structural properties such as its bending, major groove widening and flexibility [17, 24–26]. The target DNA helix is indeed severely distorted by the IN [19, 20] and concerted integration favours specific DNA distortions with an enrichment of flexible/rigid dinucleotides at the integration site [18]. The palindromic sequence present at the LTR-LTR junctions of two LTR circles and cleaved by both PFV and HIV-1 integrases also contains a specific distribution of flexible/rigid dinucleotides that could contribute to this cleavage property or integrases [3, 4]. Therefore, we hypothesized that the sequence-dependent mechanical cost of the DNA deformation induced by IN binding could be a key contributor to the integration free energy. We applied this hypothesis at a genomic scale, and calculated the energy profiles around independent sets of HIV-1 integration sites identified in infected cells (Fig 6B). These results revealed a global decrease of deformation energy around the sites minored by large energy fluctuations within a 150 bp window, suggesting that the physical properties of DNA play a role in IN binding, and constitute a first level of IN selectivity. These properties are perturbed by the presence of a nucleosome, which could explain the energy fluctuations observed around the integration sites. Altogether, we find that even our simple mechanical model already explains a large part of the integration features observed both *in vitro* and *in vivo*, and also provides a natural explanation as to why nucleosomes modify the local distributions of integration sites. The next step will be to study how the target DNA structural and mechanical properties can conciliate both IN and nucleosome binding constraints, at the natural integration sites.

In this study, we compared HIV-1 integration sites identified in infected cells [21, 22] with actual nucleosome positions mapped experimentally along the complete cell genomes [52, 53]. Using only experimental data, we observed a clear enrichment of integration sites within nucleosomal DNA present in a chromatin landscape characterized by a lower nucleosome density (Fig 6B and S4 Fig). This low density chromatin landscape was already observed with predicted nucleosome positions [28] and is consistent with the selectivity of HIV-1 integration in actively transcribed genes characterized by a more dynamic chromatin organization [15, 21, 22]. This study is the first to show a significant peak of nucleosome occupancy centred at integration sites. This result does not imply that integration is favoured at the nucleosome dyad, but it could be explained by random integration favoured within a mono or di-nucleosome structure located in a low nucleosome occupancy environment (S5 Fig). A possible limitation of this study involves the use of nucleosome positions and integration sites identified in different cell types. However, similar correlations were obtained with two sets of integration sites and three nucleosome maps (two determined *in vivo* and one predicted). This strengthens our conclusions and suggests that they do not depend on the cell type.

Altogether, these genomic studies confirm that both target DNA structural properties probed during IN binding and DNA wrapping within nucleosomes are two major determinants of HIV-1 integration selectivity. Further work remains to be done to define the role of additional parameters and to narrow the gap between *in vitro* and *in cellulo* approaches.

## Supporting Information

S1 Fig AFM analysis of nucleosome occupancy on the four selected sequences. A) PN templates assembled on the DX598014 1.2 kb fragment, end labeled with dATP biotin-streptavidin complex were visualized in air by Atomic Force Microscopy. (see experimental procedure for more details). B) The number of nucleosomes on PNs assembled on one sequence (DX598014) and at 4 ratios of assembly were counted and represented as a percentage of the total. C) The number of nucleosomes on PNs assembled on the four selected sequences and at one histone/DNA ratio (0.74 μg/1 μg) were counted (n = 120–200) and represented as a percentage of total. D) Predicted mean nucleosome number <N> on the four sequences at different chemical potential μ.
(TIF)

S2 Fig MNAse digestion products obtained on the four selected sequences, naked or chromatinized. Similarly to Fig 2, MNase digestion products obtained on naked DNA (panels DNA) or chromatinized templates (panels Nucl.) assembled at histone/DNA ratio of 0.74 μg/1 μg on the four selected sequences (CL529183, CL529481, CL528939 and DX598014), are represented by black points along the four sequences, according to their centre (X axis) and size (Y axis). To clarify this representation, only one tenth of the total MNase seq products are plotted.
(TIF)

S3 Fig *In vitro* integration into the four selected chromatinized templates. PN templates previously studied for nucleosome positioning (CL529183, CL529481, CL528939 and DX598014) were used as acceptor templates of integration. Integration assays were performed using a radiolabelled U3-SupF-U5 donor, either the IN-LEDGF/p75 complex [20] or IN alone [57] and following a protocol adapted from [58] (a) or [27] (b). Integration products were deproteinized, separated on a 1% agarose gel and revealed with a Fuji radioactivity image reader.
(TIF)

S4 Fig Nucleosome occupancy around HIV-1 integration sites. Similar study as the one presented in Fig 6B but with a different set of nucleosomes map identified in global CD4+ T-cells [53] (magenta line) Compilations are also presented along 16 kb (left panels) or 4 kb (right panels) windows centred around the integration sites.
(TIF)

S5 Fig Modelling the nucleosome landscape around native HIV-1 integration sites. Mean experimental nucleosome occupancy profiles (orange [52] and red [53]) around integration sites [21] indicate that integration is not random and occurs preferentially in a region of locally higher nucleosome occupancy. The "triangular" pattern and its size are consistent with an integration that occurs equiprobably within a dinucleosome flanked by less occupied an randomly phased nucleosome arrays: A) "toy model" of chromatin around integration sites: individual profiles around integration sites are composed of a central dinucleosome pattern (of size 322 bp, ie with a linker size of 30 bp) bordered by randomly and less spaced nucleosomes (of size 146 pb). B) Comparison between the experimental (red [53] and orange [52]) and the “toy model” mean nucleosome occupancy profiles when considering equiprobale integration within a dinucleosome (black, solid curve) or within a mononucleosme (of size 146 bp) (black, dashed curve).
(TIF)

S1 Table Number of integration sites identified for each sequence and experimental conditions. (TIF)

S2 Table Pearson correlation between integration sites profiles identified in the four selected sequences and IN binding preference ρIN(s) based on the DNA deformation energy. Integration sites and binding preference profiles were preliminary smoothed by a 10 bp sliding window.
(TIF)
