# Supplementary figures and images for: DNA Physical Properties and Nucleosome Positions Are Major Determinants of HIV-1 Integrase Selectivity
Source: PLoS One. 2015 Jun 15;10(6):e0129427. doi: 10.1371/journal.pone.0129427 (PMC4468133; doi:10.1371/journal.pone.0129427)

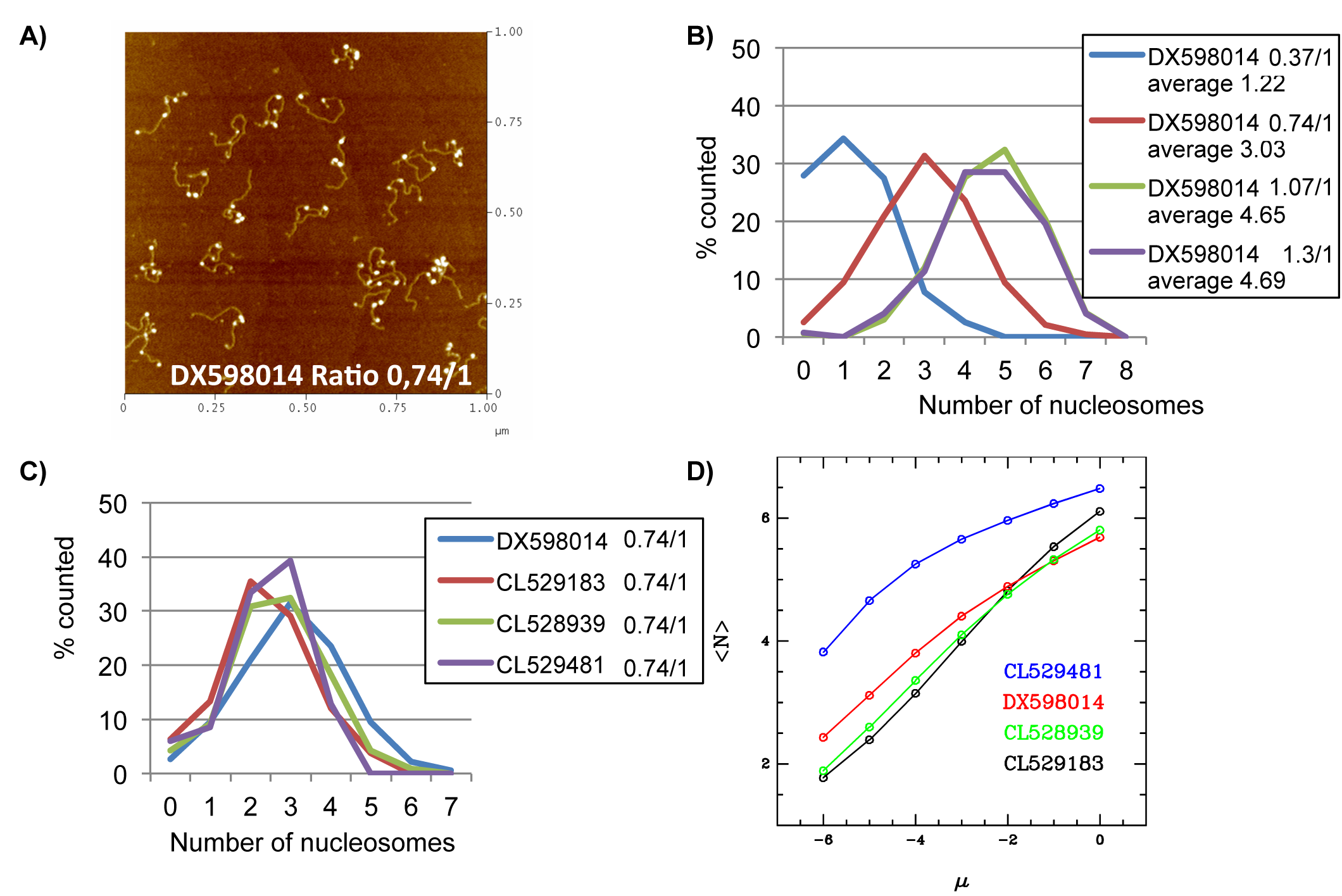

Supplement: S1 Fig — A) PN templates assembled on the DX598014 1.2 kb fragment, end labeled with dATP biotin-streptavidin complex were visualized in air by Atomic Force Microscopy. (see experimental procedure for more details). B) The number of nucleosomes on PNs assembled on one sequence (DX598014) and at 4 ratios of assembly were counted and represented as a percentage of the total. C) The number of nucleosomes on PNs assembled on the four selected sequences and at one histone/DNA ratio (0.74 μg/1 μg) were counted (n = 120–200) and represented as a percentage of total. D) Predicted mean nucleosome number on the four sequences at different chemical potential μ. (TIF) [file pone.0129427.s001.tif]

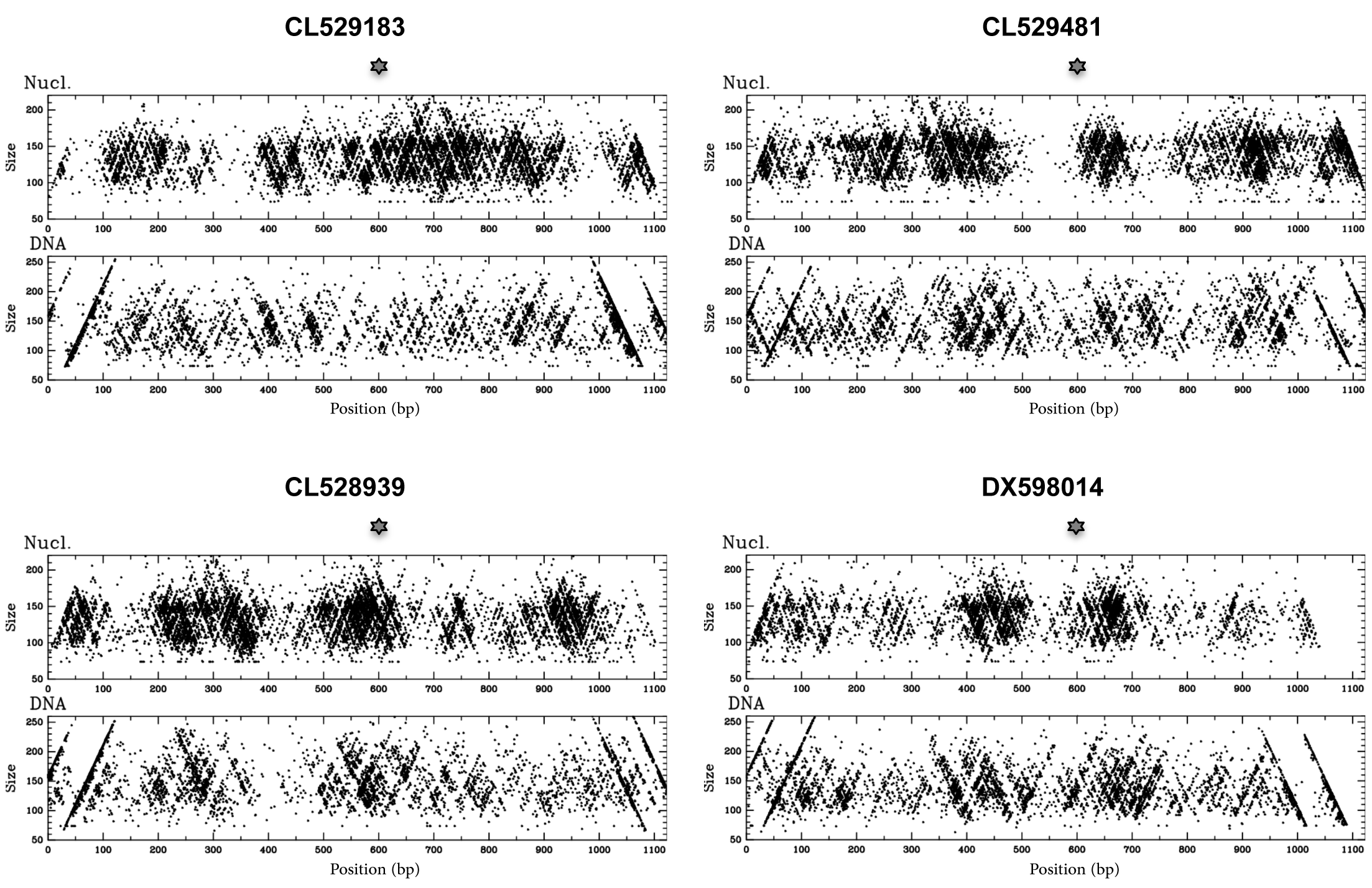

Supplement: S2 Fig — Similarly to Fig 2, MNase digestion products obtained on naked DNA (panels DNA) or chromatinized templates (panels Nucl.) assembled at histone/DNA ratio of 0.74 μg/1 μg on the four selected sequences (CL529183, CL529481, CL528939 and DX598014), are represented by black points along the four sequences, according to their centre (X axis) and size (Y axis). To clarify this representation, only one tenth of the total MNase seq products are plotted. (TIF) [file pone.0129427.s002.tif]

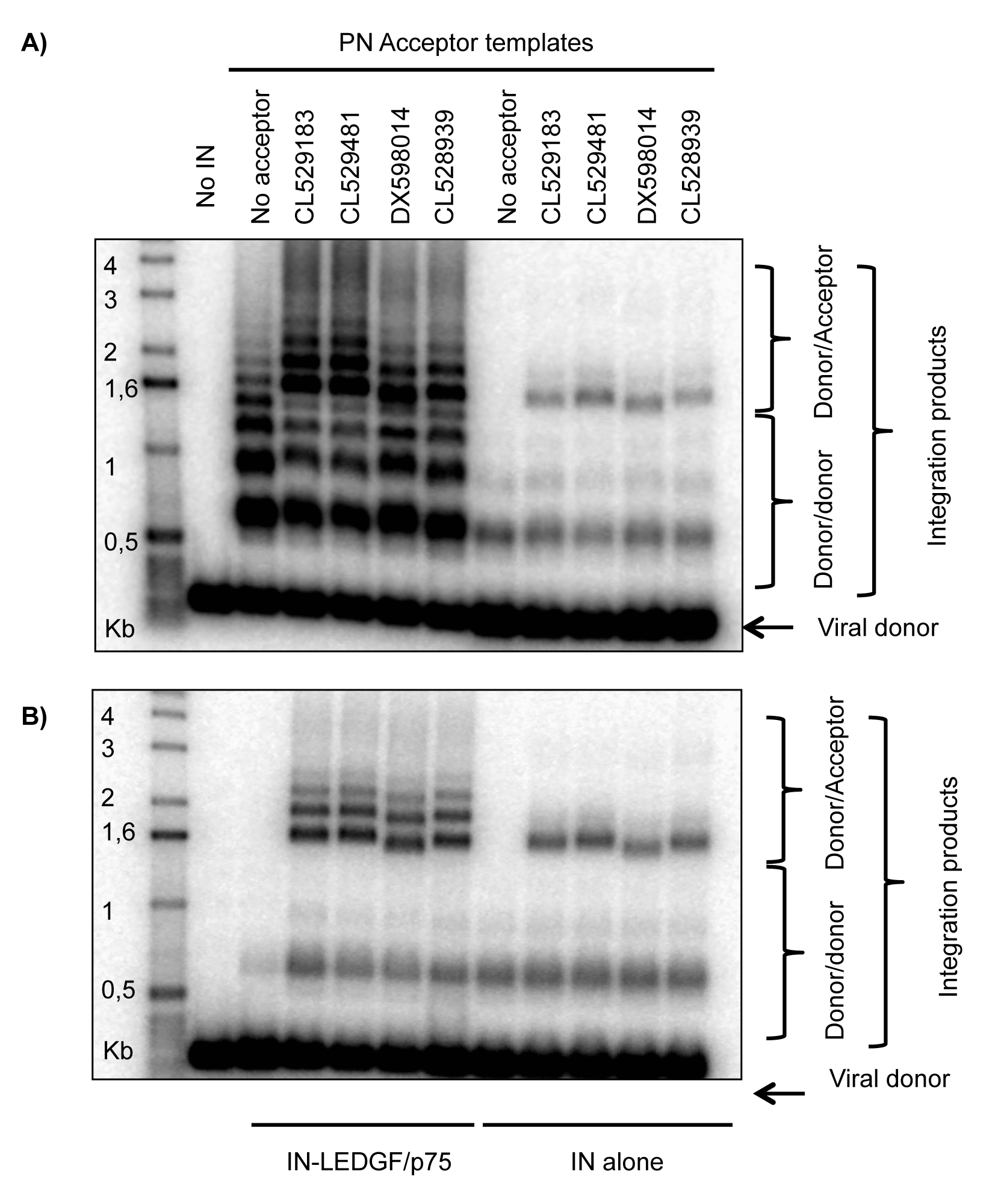

Supplement: S3 Fig — PN templates previously studied for nucleosome positioning (CL529183, CL529481, CL528939 and DX598014) were used as acceptor templates of integration. Integration assays were performed using a radiolabelled U3-SupF-U5 donor, either the IN-LEDGF/p75 complex [20] or IN alone [57] and following a protocol adapted from [58] (a) or [27] (b). Integration products were deproteinized, separated on a 1% agarose gel and revealed with a Fuji radioactivity image reader. (TIF) [file pone.0129427.s003.tif]

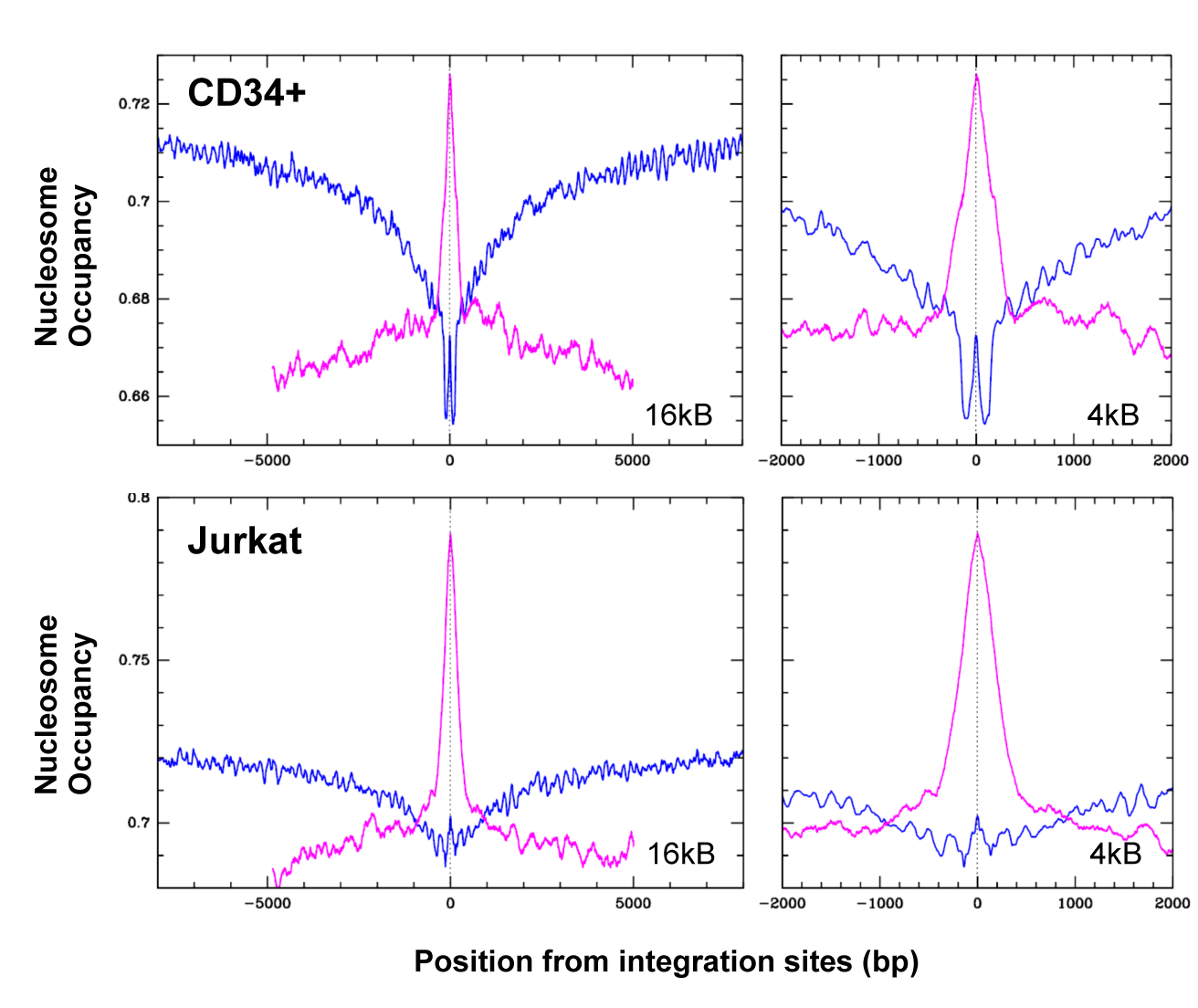

Supplement: S4 Fig — Similar study as the one presented in Fig 6B but with a different set of nucleosomes map identified in global CD4+ T-cells [53] (magenta line) Compilations are also presented along 16 kb (left panels) or 4 kb (right panels) windows centred around the integration sites. (TIF) [file pone.0129427.s004.tif]

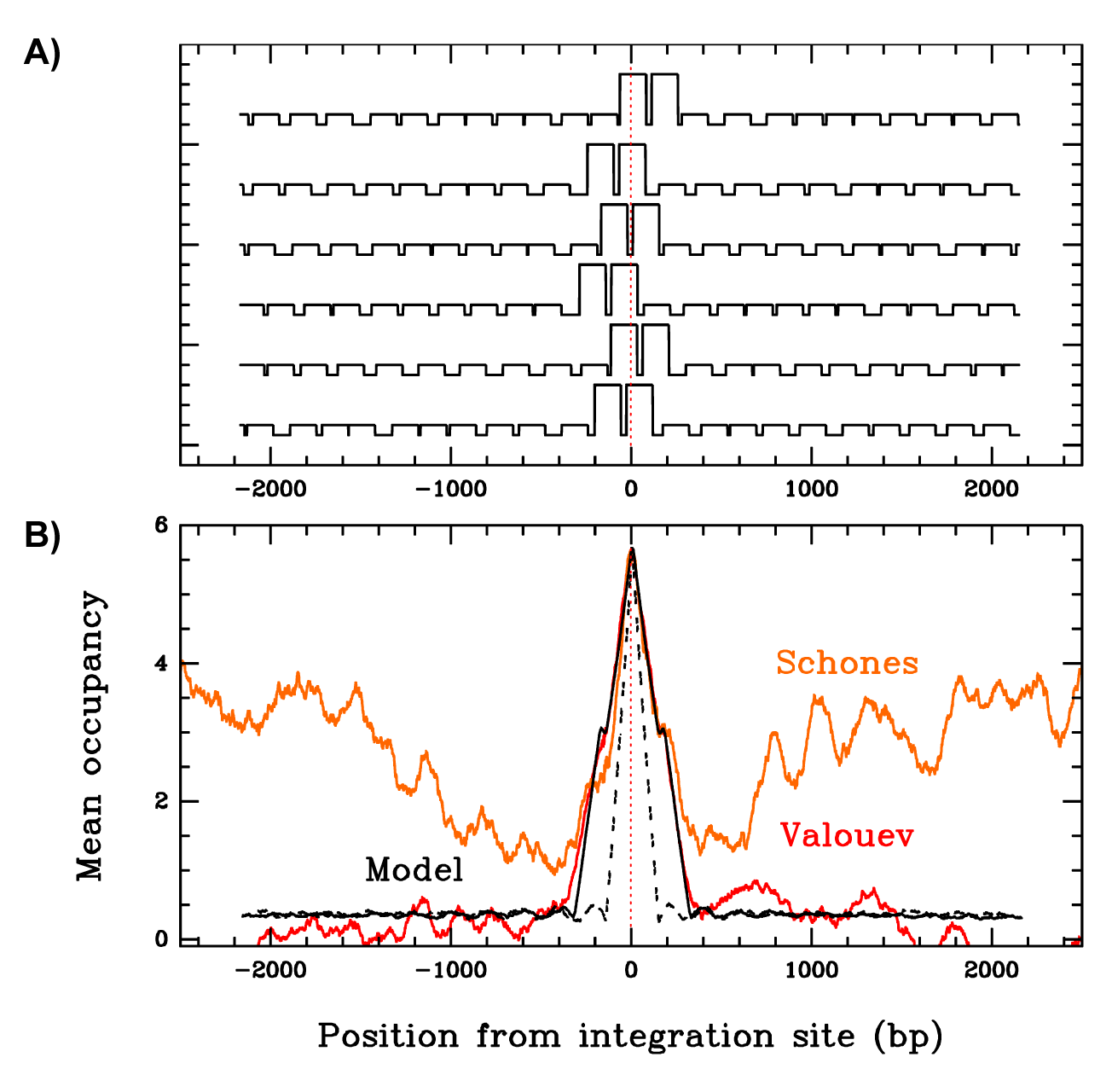

Supplement: S5 Fig — Mean experimental nucleosome occupancy profiles (orange [52] and red [53]) around integration sites [21] indicate that integration is not random and occurs preferentially in a region of locally higher nucleosome occupancy. The "triangular" pattern and its size are consistent with an integration that occurs equiprobably within a dinucleosome flanked by less occupied an randomly phased nucleosome arrays: A) "toy model" of chromatin around integration sites: individual profiles around integration sites are composed of a central dinucleosome pattern (of size 322 bp, ie with a linker size of 30 bp) bordered by randomly and less spaced nucleosomes (of size 146 pb). B) Comparison between the experimental (red [53] and orange [52]) and the “toy model” mean nucleosome occupancy profiles when considering equiprobale integration within a dinucleosome (black, solid curve) or within a mononucleosme (of size 146 bp) (black, dashed curve). (TIF) [file pone.0129427.s005.tif]

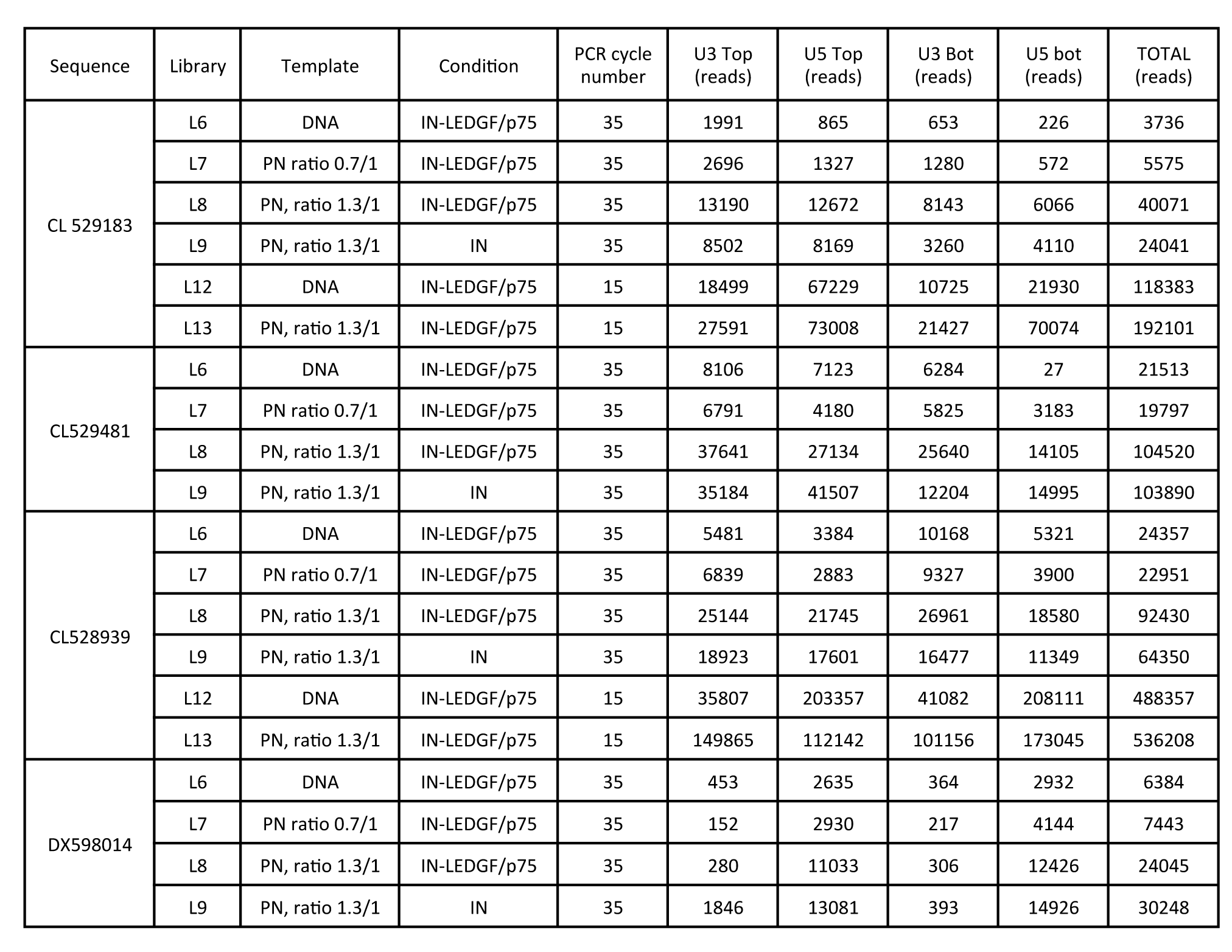

Supplement: S1 Table — (TIF) [file pone.0129427.s006.tif]

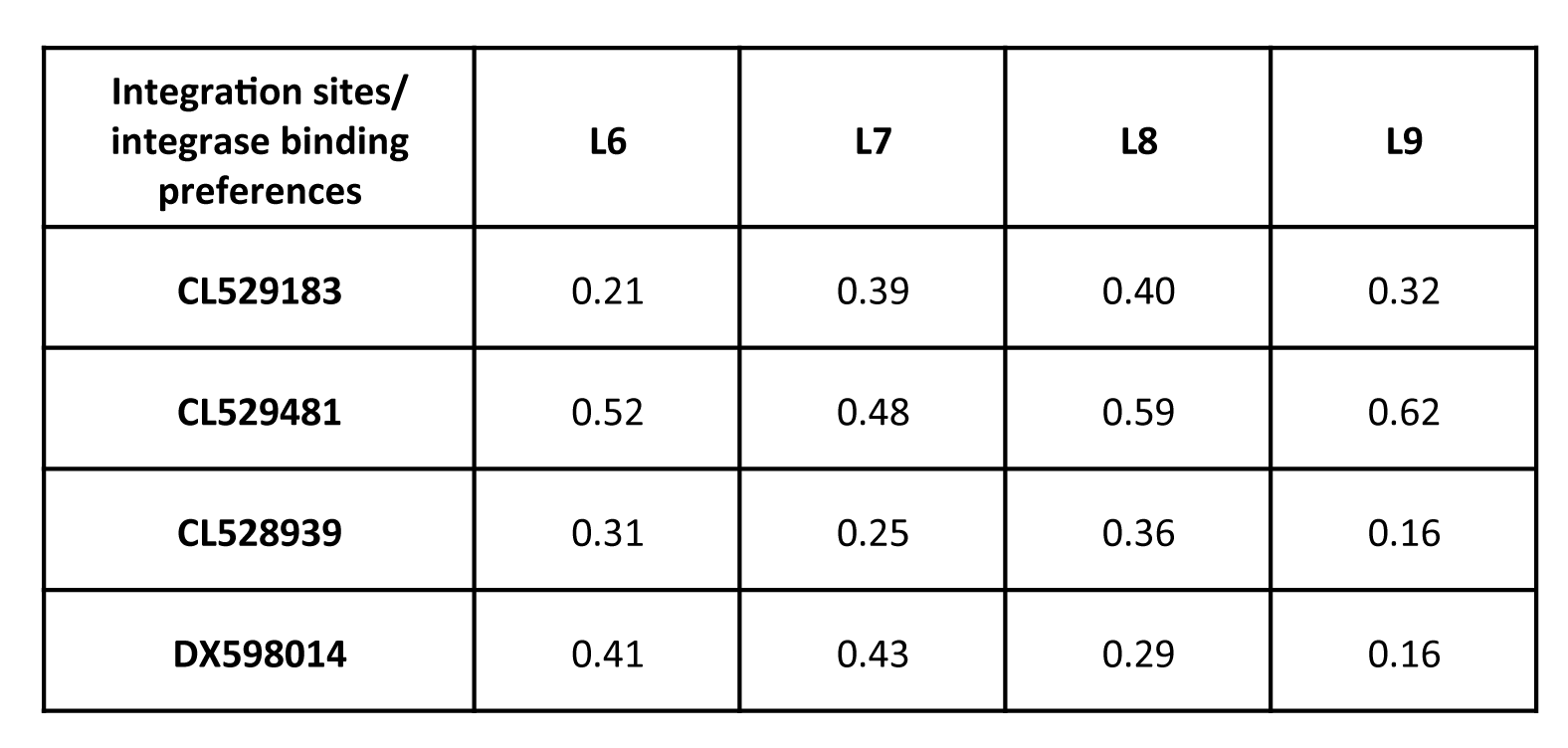

Supplement: S2 Table — Integration sites and binding preference profiles were preliminary smoothed by a 10 bp sliding window. (TIF) [file pone.0129427.s007.tif]
